# Comparison of associations of intake of ultra-processed and non-ultra-processed whole-grain foods with cardiometabolic risk measures in Australian and US adults

**DOI:** 10.1007/s00394-026-03942-8

**Published:** 2026-03-17

**Authors:** Elissa J. Price, Mengxi Du, Eden M. Barrett, Nicola M. McKeown, Marijka J. Batterham, Fang Fang Zhang, Eurídice Martínez-Steele, Eleanor J. Beck

**Affiliations:** 1 Faculty of Medicine and Health, School of Health Sciences, University of New South Wales, Sydney, NSW Australia; 2 Department of Epidemiology, Harvard T.H. Chan School of Public Health, Boston, MA USA; 3 Department of Nutrition, Harvard T.H. Chan School of Public Health, Boston, MA USA; 4 Clinical and Translational Epidemiology Unit and Division of Gastroenterology, Massachusetts General Hospital and Harvard Medical School, Boston, MA USA; 5 Faculty of Medicine and Health, The George Institute for Global Health, University of New South Wales, Sydney, NSW Australia; 6 Department of Health Sciences, Programs in Nutrition, Sargent College of Health and Rehabilitation Sciences, Boston University, Boston, MA USA; 7 Faculty of Science, Medicine, and Health, School of Medical, Indigenous, and Health Sciences, University of Wollongong, Wollongong, NSW Australia; 8 Friedman School of Nutrition Science and Policy, Tufts University, Boston, MA USA; 9 Center for Epidemiological Studies in Health and Nutrition, University of Sao Paulo, Av. Dr. Arnaldo 715, Sao Paulo, 01246-904 Brazil

**Keywords:** NNPAS, NHANES, Whole grain, Nova, Ultra-processed food, Non-ultra-processed food, Cardiometabolic risk

## Abstract

**Purpose:**

Evidence suggests total ultra-processed food (UPF) consumption increases disease risk. As whole grains are health protective, their inclusion in UPF definitions warrants consideration. We aimed to quantify Australian and US whole-grain consumption by level of food processing and compare associations on cardiometabolic risk measures.

**Methods:**

A cross-sectional analysis of Australian (NNPAS 2011-12)(n = 7735) and US (NHANES 2015-18)(n = 8343) nationally representative 2d intake data. The Nova classification system determined levels of processing. Mean and median whole-grain intakes were estimated by processing level, and regression models were used to explore associations across tertiles for total, non-UPF (Nova 1-3) and UPF (Nova 4) whole-grain intakes with cardiometabolic risk measures.

**Results:**

Adults median total whole-grain intake was 34.3 g/10 MJ/d (Australia) and 11.6 g/10 MJ/d (US). Mean whole-grain intake from UPF sources was higher in the US (71.0%) compared to Australia (48.9%). In Australia and the US, respectively, tertiles of non-UPF whole-grain intake were inversely associated with body weight (*p* = 0.02; *p* = 0.002), BMI (*p* = 0.001; *p* < 0.0001), waist circumference (*p* = 0.02; *p* = 0.001), waist-to-height ratio (WHR) (*p* = 0.003; *p* = 0.0001), and C-reactive protein (CRP) (*p* = 0.02; *p* = 0.0003), and for fasting plasma glucose (*p* = 0.002) in Australia only. UPF whole-grain intake was inversely associated with WHR (*p* = 0.04; *p* = 0.03) and diastolic blood pressure (*p* = 0.01; *p* = 0.03), as well as waist circumference (*p* = 0.0496) in Australia, and BMI (*p* = 0.03) and CRP (*p* = 0.02) in the US. Non-UPF whole-grain sources had stronger inverse associations than UPF sources.

**Conclusions:**

All whole grain foods should be promoted in public health and consumer messaging with emphasis on less processed sources given their greater observed benefit to cardiometabolic risk measures.

**Supplementary Information:**

The online version contains supplementary material available at 10.1007/s00394-026-03942-8.

## Introduction

A whole grain is typically defined as the intact, ground, cracked or flaked caryopsis including that the components—the starchy endosperm, germ and bran—are present in the same relative proportion as they exist in the intact caryopsis [1–3]. Whole-grain intake is associated with reduced risk of cardiovascular disease (CVD) and total cancer, mortality from all causes, and diabetes [4]. The Australian Dietary Guidelines (ADG) [5] and the Dietary Guidelines for Americans (DGA) [6] provide evidence-based nutrition and dietary recommendations which emphasise the importance of whole grains to include every day and state “healthy dietary patterns include whole grains” and that “at least half of total grains should be whole grains” [5, 6].

The Nova food classification system defines ultra-processed foods (UPF) as “industrial formulations typically with five or more and usually many ingredients” compared to less processed foods that range from edible parts of plants, animals, fungi, algae, and water to relatively simple products made by adding sugar, oil, salt, or other basic ingredients [7]. Extensive evidence has linked consumption of UPFs with negative health outcomes, including an increased risk of obesity and cardiometabolic conditions [8].

Grain foods including whole-grain varieties are inedible without some degree of processing to facilitate safe consumption by removing small percentages of contaminants such as microbial and heavy metals [9]. The HEALTHGRAIN whole grain definition reflects this in describing that up to 2% of the outer grain layer may be removed during processing to ensure food safety [9]. A higher degree of processing of whole-grain foods is often required to improve nutrient bioavailability [9]. Moreover, such processing is common because traditional whole-grain products have frequently faced limited acceptance in modern diets due to sensory preferences, accessibility issues, and a lack of consumer awareness [9, 10]. Whole-grain foods are represented across various categories of the Nova food classification system, from rolled oats, brown rice, and homemade bread that are considered less processed to industrially produced breads and ready-to-eat breakfast cereals, which contain substances of rare culinary use or cosmetic additives, that are considered ultra-processed. Key elements of differentiation between non-ultra-processed and ultra-processed is the presence of additives, such as emulsifiers in whole-grain foods, added for reasons such as textural improvements and to extend the shelf stability of bread (for example).

Despite links of the Nova UPF category with poor health outcomes, nuances exist in the evidence at the subgroup level. Whole-grains foods within the UPF category, specifically dark or whole-grain breads, have more recently been associated with health protective associations including a 4% risk reduction for type 2 diabetes (T2D), 2% for cardiovascular disease, 7% for stroke, and 3% for risk of multimorbidity of cancer and cardiometabolic diseases [11–13]. Research also suggests that associations between UPF intake and cardiometabolic risk measures remain relatively unchanged when excluding these foods from the UPF category [14, 15], however, it has not previously evaluated if differences exist between the health impacts of ultra-processed versus less processed whole-grain foods directly. It is important to investigate this as breads and ready-to-eat cereals are primary sources of whole-grain intake both in Australia and the United States (US) [16, 17]. In addition, both populations currently do not meet the daily intake target of 48 g of whole grains (otherwise considered as three 1-oz servings or approximately 90 g of whole-grain food per day) as recommended in the DGA and by the Grains and Legumes Nutrition Council (a not-for-profit health promotion charity in Australia) [6, 18–20].

Therefore, this research aimed to investigate the contribution of ultra-processed whole-grain intake to existing low intakes and understand if recommendations of ultra-processed whole grains should differ from their non-ultra-processed counterparts based on health-related impacts. To address this research question, we quantified the consumption of whole grains *by level of processing* according to Nova, both in the US and Australia, and further compared differences in the cross-sectional associations with cardiometabolic risk measures between non-ultra-processed versus ultra-processed sources of whole grains.

## Methods

### Study design and population

This study included data from the Australian National Nutrition and Physical Activity Survey (NNPAS) 2011-12 and the National Health and Nutrition Examination Survey (NHANES) 2015-18, both nationally representative, cross-sectional surveys conducted by the Australian Bureau of Statistics (ABS) and the US Centres for Disease Control and Prevention (CDC) and the National Centre for Health Statistics (NCHS), respectively [21]. These data were chosen as research had previously identified negative health associations with increased proportions of UPF and positive associations with increased whole-grain intake [14, 15]. All methods were duplicated for both datasets except where differences between the cohorts made this not possible. Variations in methods are reported where they exist. This study includes data collected on 7735 participants from NNPAS who were aged 19 years were and older with two days of valid dietary intake data and 8343 participants from the US based on two 2-year cycles (2015-2018) of NHANES who were aged 20 years or older and also reported two days of valid dietary intake data (Supplementary Material 1 and 2). NNPAS and NHANES collected information on the non-institutionalised civilians living in respective countries. Accessibility and dissemination of Australian data are governed by Section 15 of the Census and Statistics (Information Release and Access) Determination 2018 under the Census and Statistics Act 1905. NHANES was approved by the research Ethics Review Board of NCHS and written informed consent was provided from all participants [22].

### Dietary assessment, nova, and whole-grain and refined grain intake calculations

Both the Australian NNPAS and US NHANES collected dietary intake data using two 24-hour (24-h) dietary recall assessments with the initial completed as face-to-face interviews and the follow-up via telephone. Both surveys also utilised an adapted version of the Automated Multiple Pass Method (AMPM) to assist dietary recall. In Australia, the initial recall was completed on 12,153 participants and 7735 at follow up. In the US, intake data were collected for one day for 16,147 participants and the second recall was completed with 13,666 participants. Further details of NNPAS and NHANES data collection methods and protocol can be found elsewhere [21, 23]. Australian analyses in the current study included data from the ‘Australian Health Survey: Nutrition and Physical Activity, 2011-12 expanded confidentialised unit record files (CURF)’ dataset.

The 2011-13 Australian Food and Nutrient (AUSNUT) Nova database and the NHANES 2015-18 Nova database were used in this study to categorise foods according to Nova. Nova categorises foods into one of four groups depending on the nature, extent, and purpose of the processing undergone [7]. Nova group 1 includes unprocessed or minimally processed foods that are edible parts of plants or natural foods altered by processing only to remove inedible or unwanted parts, while group 2 consists of processed culinary ingredients such as oils and sugars derived from group 1 or nature [7]. Group 3 includes manufactured processed foods made by adding sugar, oil, salt, or other group 2 substances to group 1 foods, and group 4 comprises ultra-processed foods—manufactured foods that include substances of rare culinary use and/or cosmetic additives such as colours, flavours, and others [7]. Examples of whole-grain food items categorised according to the Nova classification system are provided in Table 1**.**

**Table 1:** Examples of whole-grain food items categorised according to the Nova Classification System

Nova 1	Nova 2	Nova 3	Nova 4
Wheat flour Rye flour Steel cut oats Pasta Rice (any fresh variety) Barley Corn Polenta Buckwheat	NA	Most home-made/fresh unpackaged bread	Mass produced packaged bread RTE-cereals ( for example, weet-bix) Quick-cook porridge Ready-to-heat brown rice Store bought muesli Cereal and muesli bars Savoury biscuits Popcorn Corn chips Oat milk

In the 2011-13 AUSNUT Nova database foods and beverages were classified into one of the four Nova categories via assessment of the food description and ingredient list for each AUSNUT food code corresponding to NNPAS intakes, linked via these food codes, as well as from supporting AUSNUT data sources (Food Details File and Food Recipe File) [24, 25]. Similar methods were used to categorise foods consumed in NHANES 2015-18 with relevant databases including the USDA Food and Nutrition Database for Dietary Studies (FNDDS) and the USDA National Nutrient Database for Standard Reference [26, 27]. For food items deemed to be culinary preparation, or handmade recipes, Nova classification was applied at the individual ingredient level in both Australia and the US. More details on the classification of foods according to Nova are provided elsewhere [28, 29]. For the purposes of this research, the term ‘non-ultra-processed food (non-UPF)’ refers to Nova groups 1-3, and the term ‘UPF’ refers to Nova group 4.

In addition to the databases listed above, an updated version of the Australian whole-grain database [30, 31], an expansion of the AUSNUT 2011-13 database, and the USDA Food Patterns Equivalents Database (FPED) were used to calculate whole-grain intakes in Australia and the US, respectively [30]. The Australian whole-grain database provides amounts of whole grain as grams per 100 g of dry weight and was used in the present study to calculate intakes as grams per day. Whole grain as grams per 100 g of dry weight previously calculated [30] was also utilised to identify foods higher in whole grain (i.e., containing ≥ 50% and ≥ 25% whole grain) for consideration of types of whole-grain food consumption patterns across categories of Nova. The whole-grain cut-offs to define a high whole-grain food reflect the Whole Grain Initiative (WGI) global consensus definition of whole-grain foods and whole-grain front-of-pack labelling requirements, respectively [3]. The US FPED translates foods in the FNDDS into 37 Food Pattern components, including amounts (ounce equivalents) of total grains, whole grains, and refined grains per 100 g of each food [32]. Grain amounts in one ounce equivalent for flour-based grain products were defined as 16 g of flour in food items such as breads and rolls, bagels, cakes, cookies, tortillas etc. and 28.35 g of grain in grain-based food items including ready-to-eat cereals, barley, buckwheat, pasta, popcorn etc. as recommended in FPED [32]. Additional calculations adjusting for water content were completed to determine dry weights of whole grain in grams per 100 g [32],and the same cut off values (≥ 50% and ≥ 25% whole grain).

To account for differences in total dietary intake by age and sex, whole-grain intakes were also adjusted for energy intake and reported as grams per 10 MJ per day (g/10 MJ/d). Median and mean whole grain consumption, and interquartile range (IQR), were calculated at the population level for each category of whole grain intake (total, ≥ 25% WG, and ≥ 50% WG) and by level of processing according to Nova. This was additionally completed for refined grain consumption, which was also reported as absolute g/10 MJ/d and calculated to consider the relevance of proportions of whole- and refined- grain in sensitivity analyses. Refined grain data was readily available in FPED for the US as ounce equivalents per 100 g of food and therefore required conversion to grams as completed for whole-grain values [32]. For Australian data, the updated Australian whole-grain database was used to calculate refined grain intake. Using the database, refined grain for non-whole-grain ingredient items was calculated as 100 g minus the grams of whole grain based on dry weight per 100 g and then adjusted for moisture. For items considered a recipe, the same approach was applied for each ingredient with use of the Food Details File and Food Recipe File [24, 25]. For non-whole-grain items not considered a recipe and yet were not a single ingredient, foods were considered at the nutrient level using AUSNUT 2011-13 database to calculate grain content, or they were matched to similar recipe items to calculate quantities of refined grain in a food.

### Health outcomes measures

All cardiometabolic risk measure data from NNPAS 2011–12 and NHANES 2015-18, including anthropometric, blood pressure, and biochemical data were available for both Australia and the US. Specific details on the methodology of measuring these outcomes for both are available elsewhere [23, 33]. In both contexts, weight (kg), height (cm), waist circumference (cm), and systolic blood pressure (SBP)(mmHg) and diastolic blood pressure (DBP)(mmHg) were collected by trained interviewers recorded using a digital scale, stadiometer, metal tape measure, and an automated blood pressure monitor, respectively. Australian biochemical measure data were obtained from the National Health Measures Survey (NHMS) 2011-12 where biomarkers for chronic disease and nutrition status were collected using blood and spot urine samples from a subset of participants in the NNPAS 2011-12 and National Health Survey 2011-12 [23]. Similarly, in NHANES, biochemical measures were collected via blood and urine samples taken at the mobile examination centre (MEC) [19]. Biochemical measures of interest for Australia and the US include total cholesterol (mmol/L), HDL-cholesterol (mmol/L), LDL-cholesterol (mmol/L), fasting triglycerides (mmol/L), fasting plasma glucose (mmol/L), glycated haemoglobin (HbA1c) (%), and C-reactive protein (CRP) (mg/L). Fewer measures were available for LDL-cholesterol, fasting triglycerides, and fasting plasma glucose as data were only obtained from participants who had fasted for 8 h or more prior to blood sample collection in Australian data or were scheduled a morning session at the MEC who had also fasted for 9 h prior, as determined by the MEC phlebotomist, prior to the blood draw for the US [21, 23].

### Statistical analysis

For both Australian and US analyses, participants were separated into ‘non consumers’ and tertiles of consumers based on grams of intake per day for each category of whole grain (total, UPF, and non-UPF), and hereafter referred to as categories of whole-grain intake. Tertile 1 indicates participants with the lowest whole-grain intake, versus tertile 3 being those with the highest. Demographic characteristics were analysed for each category of whole grain within both Australian and US datasets, where continuous variables were analysed using linear regressions with a variation of age, sex, and/or energy intake and ethnicity as confounders, and a test for linear trend was determined. Statistical significance for all tests was set to *p* < 0.05. Categorical demographics were analysed by Pearson’s χ^2^ with *p* < 0.05 representing significant results. A Bonferroni correction for multiple comparisons in characteristics analyses, thus *p* < 0.008 (0.05/6).

Multiple linear regressions assessed associations between non-consumers and tertiles of total, UPF, and non-UPF whole-grain intake with continuous anthropometric, blood pressure, and biochemical health outcomes. Analyses for intake categories used the ‘non-consumer’ group as the reference category for each (i.e., 0 g of whole-grain intake). Exploratory data analysis of the outcome variables was conducted prior to performing the regressions and fasting triglycerides, fasting plasma glucose, HbA1c, and CRP were log transformed prior to being analysed due to skewed distributions. All log-transformed outputs were back-transformed and the exponentiated beta-coefficient (β) was interpreted as a percentage change in the original scale between categories of the independent variable and calculated as 100x(e^β^-1). For example, a CRP result of 0.75 represents a 25% reduction between categories of intake as 0.75–1 = − 0.25). Regression models and covariates differed depending on the outcome of interest. Details of confounding variables for Australia [23] and the US [14, 33] are provided in Supplementary Material 3 and 4. Sensitivity analyses adjusting for refined-grain intake were completed for all outcomes, and adjustments for BMI and waist circumference (WC) were also completed for blood pressure and biochemical outcomes in both populations. The primary outcome of the paper reports the beta coefficient, 95% CI, and *p*-value for linear trend across categories of total, UPF, and non-UPF whole-grain intake for each cardiometabolic risk measure. Statistical significance at the tertile of intake level is indicated using the 95% CI results. Statistical significance for all tests was set to *p* < 0.05.

Participants < 19 years of age in Australia (n = 2812) and participants < 20 years of age (n = 7509) in the US were excluded (respective ages based on the definition of ‘adults’ in each survey). Additionally, those who recorded only 1 day of dietary intake data (US n = 2945; Aus n = 4418), and those with 2d of dietary intake who under- or over- reported energy intakes based on a 99.7% confidence interval for both Australia and the US were also excluded. Each health outcome was analysed individually and therefore participants were excluded from analyses if the outcome or at least one covariate from respective regression models were missing for both Australia and the US (range of final participants included: n = 1853 to n = 5218 and n = 2270 to n = 7006, respectively). Participants taking prescribed lipid or cholesterol lowering medication (Australia n = 557 and US n = 2298) were excluded from the total cholesterol, LDL-cholesterol, triglycerides, and CRP analyses. All analyses of Australian data were completed using Stata (StataCorp Stata Statistical Software: Release 18, 2023) and US analyses using SAS (Version 9.4, SAS Institute Inc, Cary NC). Complex survey design methods were applied for both datasets, incorporating sampling and replicate weights to allow for generalisation of results to the Australian population and the US civilian resident noninstitutionalised population at the time of the respective surveys. Australian analyses incorporated the use of two separate sets of weighting to maximise population size and increase result precision for each measure. Thus, anthropometric and blood pressure measures applied person-level weights, while biochemical measures utilised biochemical weights [34]. For all outcomes in the US analyses, dietary day 1 and 2 sample weights were used [35].

## Results

### Characteristics and nutrient intakes of study participants

Demographic characteristics of Australian and US adult survey populations have been reported elsewhere [14, 34]. Of note, 32.8% of individuals were non-consumers of whole grains in the US sample compared to 13.8% in the Australian sample (Supplementary Materials 5 and 6 include respective results). Trends in whole-grain intake by sex were similar in Australia and the US where proportions were relatively even among non-consumers (Australia 56.5% male and 43.5% female; US 51.8% and 48.2%, respectively) and those in the highest tertile of total whole-grain intake (Australia 51.1% male and 49.9% female) with slightly more females in the highest tertile of intake in the US (45.7% male and 54.3% female). The largest difference was evident in the highest tertile of non-UPF whole-grain consumers in the US with 60.3% female and 39.7% male. Mean age patterns were similar. The mean age of non-consumers was 43.9 (0.9) years in Australia and 45.2 (0.6) years in the US. For the highest tertile of whole-grain consumers, mean age was 51.1 (0.6) years in Australia and 50.5 (0.9) years in the US.

### Whole and refined grain consumption (total and by level of processing)

Similar to Australia, majority of intake in the US was from foods higher in whole grain (≥ 25% and ≥ 50% whole-grain containing) with median intakes of 8.4 g/10 MJ/d and 0 g/10 MJ/d, respectively, but the proportion of ≥ 50% whole-grain containing was lower than in Australia. Comparatively, a larger proportion of total whole-grain intake was from UPF sources (71.0%) versus non-UPF sources (29.0%) based on mean intakes also. Lower median intakes were also similarly found for higher whole-grain containing foods (≥ 25% and ≥ 50%) in the US yet were still largely comprised from UPF sources (68.1% and 74.8%, respectively) compared to non-UPF sources (31.9% and 25.2%, respectively) based on mean intakes. Median total intake from all sources of refined grain intake in the US was 81.5 g/10 MJ/d. Proportions of refined grain intake from UPF and non-UPF sources were similar to that of refined grains in Australian data based on mean intakes (70.5% and 29.6%, respectively).

Median total whole and refined grain consumption for all adults with 2d of intake in NHANES 2015-18 (11.6 g/10 MJ/d and 81.5 g/10 MJ/d, respectively) was lower than that for all adults with 2d of intake in NNPAS 2011-12 (34.3 g/10 MJ/d and 104.8 g/10 MJ/d, respectively) (Table 2). Intakes by level of processing according to Nova is also illustrated in Table 2. No grain foods are classified within the Nova 2 category of processed culinary ingredients. The majority of whole-grain intake in Australia was from foods higher in whole grain (≥ 25% and ≥ 50% whole-grain containing) with median intakes of 32.1 g/10 MJ/d and 22.5 g/10 MJ/d, respectively. Total whole-grain intakes for all categories of whole grain were relatively evenly comprised of non-UPF and UPF whole-grain sources (approximately 50/50 based on mean values). Median total intake from all sources of refined grain intake was 104.8 g/10 MJ/d in Australian adults, where refined grain intake was comprised more of UPF sources (62.9%) than non-UPF sources (37.1%) (mean intake).

**Table 2:** Median and mean energy-adjusted whole-grain and refined grain consumption according to Nova for adults in NNPAS 2011-12 and NHANES 2015-18 with 2d of dietary recall (n = 6053 and n = 8343, respectively)

	Total	Nova 1	Nova 3	Nova 4	Percentage of ultra-processed	Percentage of non-ultra-processed
*Median g/10 MJ/day (IQR)*						
NNPAS 2011-12 (Australia)						
Whole grains	34.3 (9.9–66.6)	0 (0–0)	0 (0–19.4)	10.8(0–33.6)	–	–
≥ 25% WG	32.1 (6.1–64.5)	0 (0–0)	0 (0–19.1)	7.3 (0–31.5)	–	–
≥ 50% WG	22.5 (0–54.6)	0 (0–0)	0 (0–0)	0 (0–27.7)	–	–
Refined grains	104.8(69.3–144.6)	0 (0–12.5)	18.1 (0–38.3)	63.9 (35.5–97.8)	–	–
NHANES 2015-18 (United States)						
Whole grains	11.6 (0–35.1)	0	–	6.9 (0–25.0)	–	–
≥ 25% WG	8.4 (0–32.3)	0	–	0 (0–22.0)	–	–
≥ 50% WG	0 (0–21.2)	0	–	0 (0–14.3)	–	–
Refined grains	81.5 (50.8–121.6)	13.4 (0–38.9)	0	56.4 (31.0–89.3)	–	–
*Mean g/10 MJ/day (SE)*						
NNPAS 2011-12 (Australia)						
Whole grains	43.5(0.5)	9.7(0.3)	12.6(0.3)	21.3(0.4)	48.9%	51.1%
≥ 25% WG	41.6(0.5)	9.7(0.3)	12.4(0.3)	19.6(0.4)	47.0%	53.0%
≥ 50% WG	34.1(0.5)	9.7(0.3)	7.2(0.2)	17.2(0.3)	50.5%	49.4%
Refined grains	110.9(0.8)	16.4(0.5)	24.8(0.4)	69.8(0.6)	62.9%	37.1%
NHANES 2015-18 (United States)						
Whole grains	23.6 (31.6)	6.8 (20.4)	–	16.7 (23.8)	71.0%	29.0%
≥ 25% WG	21.4 (31.0)	6.8 (20.4)	–	14.6 (23.0)	68.1%	31.9%
≥ 50% WG	14.1 (24.2)	3.6 (12.7)	–	10.5 (20.5)	74.8%	25.2%
Refined grains	110.4 (55.2)	32.6 (43.5)	0.1 (1.6)	77.8 (47.1)	70.5%	29.6%

NHANES, National Health and Nutrition Examination Survey; NNPAS, National Nutrition and Physical Activity Survey; WG, whole grain

No whole-grain foods classified as Nova 2

### Associations of total, UPF, and non-UPF whole grain consumption with cardiometabolic risk

Across Australian **(**Table 3**)** and US **(**Table 4**)** data, all trends were inverse with higher intakes of total whole grains, UPF whole grains, and non-UPF whole grains, and lower cardiometabolic risk measures. Significant inverse associations were evident in some analyses for body weight, BMI, WC, waist-to-height ratio (WHR), fasting plasma glucose (Australia only), and CRP. Overall, associations were generally stronger for non-UPF whole-grain sources than for UPF whole-grain sources, excluding DBP, where UPF whole grains showed stronger inverse associations. For some outcomes, significant associations were found for non-UPF whole grains even when total whole grains shown no significant association. This did not occur for UPF whole-grain intake.

**Table 3:** Associations between consumption of whole grains by level of processing and cardiometabolic risk measures in Australian adults

	Non consumers	T1	T2	T3	*p* value^2^
*Whole-grain intake (g/10 MJ/d)*^*1*^* β (95% CI)*					
Body weight (kg) (n = 5218)					
Multivariate adjustment^3^					
Total WG	Ref (n 719)	0.57 (− 2.41, 3.55)	0.3 (− 2.71, 3.31)	− 1.26 (− 4.18, 1.66)	0.3732
UPF WG^4^	Ref (n 1492)	− 0.32 (− 2.44, 1.79)	− 1.77 (− 3.78, 0.23)	− 0.95 (− 2.74, 0.85)	0.1352
Non- UPF WG^4^	Ref (n 2352)	0.001 (− 1.81, 1.82)	− 0.1 (− 1.87, 1.66)	− 2.18 (− 3.99, − 0.36)	0.0245
RG adjustment^3,5^					
Total WG	Ref	0.63 (− 2.29, 3.55)	− 0.2(− 3.17, 2.78)	− 2.1(− 5.05, 0.85)	0.1231
UPF WG^4^	Ref	− 0.1 (− 2.16, 1.96)	− 1.98 (− 3.97, 0.01)	− 1.5 (− 3.29, 0.3)	0.0299
Non-UPF WG^4^	Ref	− 0.12 (− 1.95, 1.71)	− 0.6 (− 2.4, 1.2)	− 3.0 (− 4.87, − 1.13)	0.0021
BMI (kg/m^2^) (n = 5189)					
Multivariate adjustment^3^					
Total WG	Ref (n 718)	0.0004 (− 0.9, 0.9)	− 0.31 (− 1.21, 0.59)	− 0.84 (− 1.7, 0.03)	0.0409
UPF WG^4^	Ref (n 1487)	0.01 (− 0.69, 0.71)	− 0.51 (− 1.09, 0.08)	− 0.36 (− 0.96, 0.23)	0.0874
Non- UPF WG^4^	Ref (n 2341)	− 0.42 (− 0.99, 0.15)	− 0.42 (− 0.92, 0.08)	− 0.99 (− 1.56, − 0.41)	0.0014
RG adjustment^3,5^					
Total WG	Ref	0.02 (− 0.86, 0.9)	− 0.44 (− 1.34, 0.46)	− 1.07(− 1.95, − 0.18)	0.0100
UPF WG^4^	Ref	0.07 (− 0.6, 0.75)	− 0.56 (− 1.14, 0.02)	− 0.51 (− 1.11, 0.09)	0.0231
Non-UPF WG^4^	Ref	− 0.45 (− 1.03, 0.12)	− 0.56 (− 1.08, − 0.04)	− 1.21 (− 1.81, − 0.62)	0.0001
Waist circumference (cm) (n = 5171)					
Multivariate adjustment^3^					
Total WG	Ref (n 714)	− 0.16 (− 2.2, 1.88)	− 0.5 (− 2.63, 1.63)	− 1.91 (− 3.98, 0.17)	0.0726
UPF WG^4^	Ref (n 1476)	0.44 (− 1.29, 2.17)	− 1.47 (− 2.91, − 0.03)	− 0.92 (− 2.4, 0.58)	0.0496
Non- UPF WG^4^	Ref (n 2335)	− 1.09 (− 2.52, 0.35)	− 0.87 (− 2.45, 0.72)	− 1.99 (− 3.41, − 0.56)	0.0166
RG adjustment^3,5^					
Total WG	Ref	− 0.13 (− 2.13, 1.88)	− 0.87 (− 2.98, 1.25)	− 2.48(− 4.6, − 0.35)	0.0189
UPF WG^4^	Ref	0.58 (− 1.12, 2.28)	− 1.62 (− 3.06, − 0.19)	− 1.29 (− 2.81, 0.23)	0.0133
Non- UPF WG^4^	Ref	− 1.16 (− 2.61, 0.29)	− 1.22 (− 2.81, 0.38)	− 2.56 (− 4.01, − 1.11)	0.0017
Waist- to- height ratio (n = 5143)					
Multivariate adjustment^3^					
Total WG	Ref (n 712)	− 0.004 (− 0.01, 0.007)	− 0.007 (− 0.02, 0.004)	− 0.02 (− 0.03, − 0.004)	0.0074
UPF WG^4^	Ref (n 1468)	0.004 (− 0.007, 0.01)	− 0.008 (− 0.02, 0.0001)	− 0.006 (− 0.02, 0.003)	0.0355
Non- UPF WG^4^	Ref (n 2342)	− 0.01 (− 0.02, − 0.003)	− 0.009 (− 0.02, − 0.003)	− 0.01 (− 0.02, − 0.006)	0.0032
RG adjustment^3,5^					
Total WG	Ref	− 0.003 (− 0.01, 0.007)	− 0.009 (− 0.02, 0.003)	− 0.02 (− 0.03, − 0.007)	0.0021
UPF WG^4^	Ref	0.004 (− 0.006, 0.01)	− 0.009 (− 0.02, − 0.0005)	− 0.008 (− 0.02, 0.001)	0.0126
Non− UPF WG^4^	Ref	− 0.01 (− 0.02, − 0.003)	− 0.01 (− 0.02, − 0.002)	− 0.02 (− 0.03, − 0.008)	0.0005
Systolic blood pressure (mmHg) (n = 4964)					
Multivariate adjustment^6^					
Total WG	Ref (n 691)	0.22 (− 2.14, 2.58)	− 0.43 (− 2.71, 1.86)	− 0.33 (− 2.44, 1.78)	0.6139
UPF WG^4^	Ref (n 1430)	0.63 (− 1.17, 2.43)	− 0.33 (− 1.69, 1.03)	− 0.13 (− 1.56, 1.29)	0.4869
Non-UPF WG^4^	Ref (n 2246)	− 0.35 (− 1.7, 1.0)	− 0.62 (− 2.13, 0.9)	− 0.59 (− 2.21, 1.02)	0.4513
RG adjustment^3,5^					
Total WG	Ref	0.2 (− 2.17, 2.56)	− 0.23 (− 2.52, 2.06)	− 0.02 (− 2.2, 2.15)	0.8792
UPF WG^4^	Ref	0.57 (− 1.21, 2.34)	− 0.26 (− 1.6, 1.09)	0.04 (− 1.42, 1.51)	0.7281
Non- UPF WG^4^	Ref	− 0.31 (− 1.67, 1.05)	− 0.45 (− 1.97, 1.07)	− 0.33 (− 2.0, 1.34)	0.6847
BMI adjustment^6,7^					
Total WG	Ref	0.02 (− 2.07, 2.48)	− 0.27 (− 2.47, 1.93)	0.06 (− 2.01, 2.13)	0.9280
UPF WG^4^	Ref	− 0.1 (− 1.42, 1.23)	− 0.41 (− 1.94, 1.11)	− 0.12 (− 1.74, 1.51)	0.7662
Non-UPF WG^4^	Ref	0.6 (− 1.14, 2.33)	− 0.09 (− 1.45, 1.27)	0.04 (− 1.39, 1.47)	0.8076
WC adjustment^6,8^					
Total WG	Ref	0.18 (− 2.12, 2.47)	− 0.36 (− 2.56, 1.83)	− 0.03 (− 2.08, 2.02)	0.8378
UPF WG^4^	Ref	0.49 (− 1.24, 2.23)	− 0.07 (− 1.43, 1.28)	0.007 (− 1.41, 1.43)	0.7735
Non-UPF WG^4^	Ref	− 0.16 (− 1.49, 1.18)	− 0.47 (− 1.96, 1.02)	− 0.26 (− 1.86, 1.34)	0.6819
Diastolic blood pressure (mmHg) (n = 4964)					
Multivariate adjustment^6^					
Total WG	Ref (n 691)	− 1.18 (− 2.67, 0.31)	− 1.71 (− 3.22, − 0.22)	− 1.88 (− 3.31, − 0.44)	0.0074
UPF WG^4^	Ref (n 1430)	− 0.73 (− 1.97, 0.5)	− 1.47 (− 2.64, − 0.3)	− 1.59 (− 2.72, − 0.47)	0.0047
Non-UPF WG^4^	Ref (n 2246)	− 0.71 (− 1.78, 0.37)	− 0.11 (− 1.26, 1.03)	− 1.0 (− 1.94, − 0.05)	0.1459
RG adjustment^3,5^					
Total WG	Ref	− 1.18 (− 2.67, 0.31)	− 1.7 (− 3.2, − 0.2)	− 1.85 (− 3.33, − 0.36)	0.0096
UPF WG^4^	Ref	− 0.74 (− 1.96, 0.49)	− 1.47 (− 2.64, − 0.29)	− 1.58 (− 2.7, − 0.46)	0.0045
Non-UPF WG^4^	Ref	− 0.7 (− 1.77, 0.37)	− 0.1 (− 1.23, 1.03)	− 0.98 (− 1.95, − 0.006)	0.1644
BMI adjustment^6,7^					
Total WG	Ref	− 1.2 (− 2.51, 0.12)	− 1.53 (− 2.92, − 0.14)	− 1.42 (− 2.76, − 0.08)	0.0305
UPF WG^4^	Ref	− 0.77 (− 1.87, 0.32)	− 1.19 (− 2.27, − 0.1)	− 1.4 (− 2.41, − 0.38)	0.0077
Non-UPF WG^4^	Ref	− 0.41 (− 1.44, 0.62)	0.12 (− 0.98, 1.22)	− 0.44 (− 1.37, 0.49)	0.6267
WC adjustment^6,8^					
Total WG	Ref	− 1.23 (− 2.54, 0.07)	− 1.64 (− 3.0, − 0.27)	− 1.51 (− 2.78, − 0.23)	0.0141
UPF WG^4^	Ref	− 0.9 (− 2.02, 0.22)	− 1.15 (− 2.21, − 0.09)	− 1.42 (− 2.45, − 0.4)	0.0086
Non-UPF WG^4^	Ref	− 0.46 (− 1.5, 0.57)	0.07 (1.0, 1.13)	− 0.58 (− 1.46, 0.3)	0.4232
Total cholesterol (mmol/L) (n = 2243)					
Multivariate adjustment^9^					
Total WG	Ref (n 267)	− 0.02 (− 0.24, 0.2)	− 0.01 (− 0.24, 0.22)	− 0.02 (− 0.24, 0.21)	0.9032
UPF WG^4^	Ref (n 601)	− 0.004 (− 0.22, 0.22)	0.07 (− 0.1, 0.25)	0.02 (− 0.16, 0.2)	0.5814
Non-UPF WG^4^	Ref (n 935)	− 0.05 (− 0.2, 0.11)	− 0.06 (− 0.27, 0.14)	0.02 (− 0.17, 0.21)	0.8903
RG adjustment^3,5^					
Total WG	Ref	− 0.04 (− 0.26, 0.18)	− 0.07 (− 0.3, 0.17)	− 0.1 (− 0.33, 0.14)	0.4068
UPF WG^4^	Ref	− 0.003 (− 0.22, 0.22)	0.05 (− 0.13, 0.23)	− 0.02 (− 0.2, 0.17)	0.9846
Non-UPF WG^4^	Ref	− 0.06 (− 0.21, 0.09)	− 0.1 (− 0.3, 0.1)	− 0.04 (− 0.25, 0.17)	0.6287
BMI adjustment^7,9^					
Total WG	Ref	− 0.02 (− 0.23, 0.19)	− 0.008 (− 0.24, 0.22)	− 0.01 (− 0.23, 0.21)	0.9577
UPF WG^4^	Ref	− 0.01 (− 0.23, 0.21)	0.08 (− 0.1, 0.26)	0.02 (− 0.16, 0.2)	0.5978
Non-UPF WG^4^	Ref	− 0.04 (− 0.19, 0.11)	− 0.06 (− 0.26, 0.14)	0.04 (− 0.16, 0.23)	0.7743
WC adjustment^8,9^					
Total WG	Ref	− 0.02 (− 0.23, 0.19)	− 0.009 (− 0.24, 0.22)	− 0.01 (− 0.23, 0.21)	0.9419
UPF WG^4^	Ref	− 0.01 (− 0.23, 0.2)	0.08 (− 0.09, 0.26)	0.02 (− 0.16, 0.19)	0.5865
Non-UPF WG^4^	Ref	− 0.04 (− 0.19, 0.12)	− 0.06 (− 0.25, 0.14)	0.04 (− 0.16, 0.23)	0.7784
Fasting LDL− cholesterol (mmol/L) (n = 1853)					
Multivariate adjustment^9^					
Total WG	Ref (n 223)	0.009 (− 0.18, 0.2)	0.02 (− 0.19, 0.23)	0.02 (− 0.18, 0.21)	0.8489
UPF WG^4^	Ref (n 504)	0.05 (− 0.16, 0.26)	0.03 (− 0.13, 0.19)	0.06 (− 0.12, 0.24)	0.5640
Non-UPF WG^4^	Ref (n 770)	− 0.05 (− 0.2, 0.11)	− 0.002 (− 0.19, 0.19)	0.04 (− 0.15, 0.22)	0.6085
RG adjustment^3,5^					
Total WG	Ref	− 0.01 (− 0.2, 0.19)	− 0.04 (− 0.26, 0.18)	− 0.07 (− 0.28, 0.14)	0.5059
UPF WG^4^	Ref	0.04 (− 0.17, 0.25)	0.004 (− 0.16, 0.17)	0.02 (− 0.17, 0.2)	0.9819
Non-UPF WG^4^	Ref	− 0.06 (− 0.22, 0.09)	− 0.04 (− 0.23, 0.15)	− 0.03 (− 0.23, 0.17)	0.7933
BMI adjustment^7,9^					
Total WG	Ref	0.01 (− 0.18, 0.2)	0.03 (− 0.19, 0.24)	0.03 (− 0.17, 0.23)	0.7400
UPF WG^4^	Ref	0.04 (− 0.17, 0.25)	0.03 (− 0.13, 0.19)	0.05 (− 0.13, 0.24)	0.5661
Non-UPF WG^4^	Ref	− 0.03 (− 0.19, 0.12)	0.007 (− 0.18, 0.19)	0.06 (− 0.13, 0.24)	0.4661
WC adjustment^8,9^					
Total WG	Ref	0.01 (− 0.17, 0.19)	0.02 (− 0.18, 0.23)	0.03 (− 0.17, 0.23)	0.7532
UPF WG^4^	Ref	0.03 (− 0.17, 0.23)	0.03 (− 0.12, 0.19)	0.05 (− 0.13, 0.23)	0.5764
Non-UPF WG^4^	Ref	− 0.03 (− 0.18, 0.13)	0.009 (− 0.17, 0.19)	0.06 (− 0.12, 0.24)	0.4523
HDL− cholesterol (mmol/L) (n = 2651)					
Multivariate adjustment^9^					
Total WG	Ref (n 316)	− 0.01 (− 0.08, 0.06)	0.005 (− 0.06, 0.07)	0.01 (− 0.06, 0.08)	0.6350
UPF WG^4^	Ref (n 708)	− 0.03 (− 0.09, 0.03)	0.03 (− 0.03, 0.09)	0.01 (− 0.04, 0.07)	0.2767
Non-UPF WG^4^	Ref (n 1098)	0.03 (− 0.02, 0.09)	− 0.02 (− 0.08, 0.03)	0.04 (− 0.01, 0.08)	0.4865
RG adjustment^3,5^					
Total WG	Ref	− 0.01 (− 0.08, 0.06)	0.002 (− 0.06, 0.07)	0.006 (− 0.06, 0.07)	0.7451
UPF WG^4^	Ref	− 0.03 (− 0.09, 0.03)	0.03 (− 0.03, 0.09)	0.01 (− 0.04, 0.06)	0.3097
Non-UPF WG^4^	Ref	0.03 (− 0.02, 0.09)	− 0.02 (− 0.08, 0.03)	0.03 (− 0.01, 0.08)	0.5530
BMI adjustment^7,9^					
Total WG	Ref	− 0.01 (− 0.09, 0.06)	0.0009 (− 0.07, 0.07)	0.002 (− 0.08, 0.08)	0.8633
UPF WG^4^	Ref	− 0.03 (− 0.04, 0.08)	− 0.03 (− 0.08, 0.02)	0.01 (− 0.04, 0.07)	0.2239
Non-UPF WG^4^	Ref	0.02 (− 0.04, 0.08)	− 0.03 (− 0.08, 0.02)	0.01 (− 0.04, 0.07)	0.9502
WC adjustment^8,9^					
Total WG	Ref	− 0.01 (− 0.09, 0.06)	0.003 (− 0.06, 0.07)	0.005 (− 0.07, 0.08)	0.7839
UPF WG^4^	Ref	− 0.02 (− 0.08, 0.03)	0.02 (− 0.04, 0.08)	0.02 (− 0.03, 0.08)	0.2094
Non-UPF WG^4^	Ref	0.02 (− 0.04, 0.08)	− 0.03 (− 0.09, 0.02)	0.02 (− 0.03, 0.07)	0.9546
Fasting triglycerides (mmol/L)^10^ (n = 1870)					
Multivariate adjustment^11^					
Total WG	Ref (n 228)	1.01 (0.89, 1.16)	0.98 (0.85, 1.14)	0.97 (0.85, 1.1)	0.5453
UPF WG^4^	Ref (n 512)	1.01 (0.9, 1.13)	1.06 (0.97, 1.17)	1.03 (0.94, 1.13)	0.3680
Non-UPF WG^4^	Ref (n 779)	0.99 (0.9, 1.1)	0.95 (0.85, 1.07)	0.92 (0.83, 1.01)	0.0767
RG adjustment^3,5^					
Total WG	Ref	1.02 (0.89, 1.16)	1.0 (0.86, 1.15)	0.99 (0.87, 1.14)	0.8438
UPF WG^4^	Ref	1.01 (0.9, 1.13)	1.07 (0.97, 1.18)	1.04 (0.95, 1.14)	0.2479
Non-UPF WG^4^	Ref	1.0 (0.9, 1.11)	0.97 (0.86, 1.09)	0.94 (0.84, 1.04)	0.2036
BMI adjustment^7,11^					
Total WG	Ref	1.01 (0.9, 1.15)	0.99 (0.86, 1.14)	0.99 (0.87, 1.11)	0.7287
UPF WG^4^	Ref	1.0 (0.89, 1.12)	1.07 (0.98, 1.18)	1.02 (0.93, 1.11)	0.3904
Non-UPF WG^4^	Ref	1.01 (0.91, 1.12)	0.97 (0.86, 1.08)	0.95 (0.85, 1.05)	0.2180
WC adjustment^8,11^					
Total WG	Ref	1.01 (0.89, 1.14)	0.99 (0.86, 1.13)	0.98 (0.87, 1.11)	0.6628
UPF WG^4^	Ref	0.98 (0.88, 1.1)	1.07 (0.98, 1.18)	1.01 (0.92, 1.1)	0.4254
Non-UPF WG^4^	Ref	1.02 (0.92, 1.12)	0.97 (0.86, 1.08)	0.95 (0.86, 1.05)	0.2106
Fasting plasma glucose (mmol/L)^10^ (n = 2278)					
Multivariate adjustment^12^					
Total WG	Ref (n 277)	1.0 (0.97, 1.02)	0.99 (0.96, 1.01)	0.97 (0.95, 1.0)	0.0463
UPF WG^4^	Ref (n 619)	1.0 (0.99, 1.02)	0.99 (0.97, 1.01)	1.0 (0.98, 1.02)	0.5131
Non-UPF WG^4^	Ref (n 942)	0.98 (0.96, 1.0)	0.98 (0.96, 1.0)	0.97 (0.95, 0.98)	0.0015
RG adjustment^3,5^					
Total WG	Ref	1.0 (0.97, 1.02)	0.99 (0.97, 1.01)	0.98 (0.95, 1.01)	0.1111
UPF WG^4^	Ref	1.0 (0.99, 1.02)	0.99 (0.97, 1.01)	1.0 (0.98, 1.02)	0.6400
Non-UPF WG^4^	Ref	0.98 (0.96, 1.0)	0.98 (0.96, 1.0)	0.97 (0.95, 0.99)	0.0067
BMI adjustment^7,12^					
Total WG	Ref	1.0 (0.97, 1.02)	0.99 (0.97, 1.01)	0.98 (0.96, 1.01)	0.0919
UPF WG^4^	Ref	1.0 (0.99, 1.02)	0.99 (0.98, 1.01)	1.0 (0.98, 1.02)	0.5589
Non-UPF WG^4^	Ref	0.98 (0.97, 1.0)	0.98 (0.96,1.0)	0.97 (0.96, 0.99)	0.0104
WC adjustment^8,12^					
Total WG	Ref	1.0 (0.97, 1.02)	0.99 (0.97, 1.01)	0.98 (0.96, 1.0)	0.0607
UPF WG^4^	Ref	1.0 (0.98, 1.02)	0.99 (0.98, 1.01)	1.0 (0.98, 1.01)	0.4670
Non− UPF WG^4^	Ref	0.98 (0.97, 1.0)	0.98 (0.96, 1.0)	0.97 (0.96, 0.99)	0.0092
HbA1c (%)^10^ (n = 2642)					
Multivariate adjustment^12^					
Total WG	Ref (n 313)	1.0 (0.99, 1.02)	1.0 (0.98, 1.01)	1.0 (0.98, 1.02)	0.7503
UPF WG^4^	Ref (n 705)	1.01 (0.99, 1.02)	1.0 (0.99, 1.01)	1.0 (0.99, 1.02)	0.8829
Non-UPF WG^4^	Ref (n 1095)	1.0 (0.99, 1.01)	0.99 (0.98, 1.0)	1.0 (0.99, 1.02)	0.9258
RG adjustment^3,5^					
Total WG	Ref	1.0 (0.99, 1.02)	1.0 (0.98, 1.02)	1.0 (0.98, 1.02)	0.8796
UPF WG^4^	Ref	1.01 (0.99, 1.02)	1.0 (0.99, 1.01)	1.0 (0.99, 1.02)	0.8376
Non-UPF WG^4^	Ref	1.0 (0.99, 1.01)	0.99 (0.98, 1.01)	1.01 (0.99, 1.02)	0.6717
BMI adjustment^7,12^					
Total WG	Ref	1.0 (0.99, 1.02)	1.0 (0.98, 1.01)	1.0 (0.98, 1.02)	0.8833
UPF WG^4^	Ref	1.01 (0.99, 1.02)	1.0 (0.99, 1.01)	1.0 (1.0, 1.02)	0.8633
Non-UPF WG^4^	Ref	1.0 (0.99, 1.01)	0.99 (0.98, 1.0)	1.01 (0.99, 1.02)	0.7769
WC adjustment^8,12^					
Total WG	Ref	1.0 (0.99, 1.02)	1.0 (0.98, 1.01)	1.0 (0.98, 1.02)	0.8471
UPF WG^4^	Ref	1.01 (0.99, 1.02)	1.0 (0.99, 1.01)	1.0 (0.99, 1.01)	0.8457
Non-UPF WG^4^	Ref	1.0 (0.99, 1.01)	0.99 (0.98, 1.0)	1.01 (0.99, 1.02)	0.7869
C-reactive protein (CRP)^10^ (mg/L)^10^ (n = 2243)					
Multivariate adjustment^9^					
Total WG	Ref (n 267)	0.78 (0.55, 1.1)	0.75 (0.52, 1.06)	0.76 (0.53, 1.1)	0.1279
UPF WG^4^	Ref (n 601)	0.96 (0.78, 1.17)	0.91 (0.73, 1.15)	1.03 (0.81, 1.3)	0.9233
Non-UPF WG^4^	Ref (n 935)	0.74 (0.61, 0.9)	0.78 (0.61, 1.02)	0.75 (0.6, 0.92)	0.0222
RG adjustment^3,5^					
Total WG	Ref	0.77 (0.54, 1.09)	0.73 (0.5, 1.05)	0.74 (0.5, 1.08)	0.1038
UPF WG^4^	Ref	0.96 (0.78, 1.17)	0.9 (0.71, 1.13)	1.0 (0.79, 1.28)	0.8716
Non-UPF WG^4^	Ref	0.74 (0.61, 0.89)	0.77 (0.59, 1.0)	0.72 (0.57, 0.9)	0.0139
BMI adjustment^7,9^					
Total WG	Ref	0.78 (0.58, 1.04)	0.76 (0.55, 1.05)	0.81 (0.59, 1.1)	0.1671
UPF WG^4^	Ref	0.91 (0.75, 1.11)	0.94 (0.76, 1.16)	0.98 (0.79, 1.22)	0.9463
Non-UPF WG^4^	Ref	0.79 (0.66, 0.94)	0.83 (0.65, 1.06)	0.84 (0.7, 1.01)	0.1211
WC adjustment^8,9^					
Total WG	Ref	0.78 (0.56, 1.08)	0.76 (0.53, 1.07)	0.79 (0.56, 1.12)	0.1663
UPF WG^4^	Ref	0.91 (0.74, 1.11)	0.96 (0.77, 1.19)	0.98 (0.78, 1.24)	0.9817
Non-UPF WG^4^	Ref	0.79 (0.66, 0.95)	0.82 (0.64, 1.07)	0.82 (0.68, 0.99)	0.0907

BMI, body mass index; non- UPF, non-ultra- processed food; RG, refined grain; UPF, ultra-processed food; WC, waist circumference; WG, whole grain

^1^Values reported as β coefficient (95% CI)

^2^*P*-value for linear trend. A significance is determined at *p* < 0.05

^3^Survey linear regression adjusted for age, sex, education, physical activity level, smoking status, energy intake, alcohol intake, socio- economic status, country of birth, and area remoteness

^4^Simultaneously adjusted for non-ultra-processed and ultra-processed whole-grain intake

^5^Survey linear regression adjusted for refined grain intake

^6^Survey linear regression adjusted for age, sex, education, physical activity level, smoking status, energy intake, alcohol intake, socio-economic status, country of birth, area remoteness, and Na intake

^7^Survey linear regression adjusted for BMI

^8^Survey linear regression adjusted for WC

^9^Survey linear regression adjusted for age, sex, education, physical activity level, smoking status, energy intake, alcohol intake, socio-economic status, country of birth, area remoteness, saturated fat intake, monounsaturated fat, and polyunsaturated fat intake

^10^Reported as exp(β) of the natural log transformed variable

^11^Survey linear regression adjusted for age, sex, education, physical activity level, smoking status, energy intake, alcohol intake, socio-economic status, country of birth, area remoteness, saturated fat intake, monounsaturated fat, polyunsaturated fat intake, and added sugar intake

^12^Survey linear regression adjusted for age, sex, education, physical activity level, smoking status, energy intake, alcohol intake, socio-economic status, country of birth, area remoteness, and added sugar intake

**Table 4:** Associations between consumption of whole grains by level of processing and cardiometabolic risk measures in US adults

	Non consumers	T1	T2	T3	*p* value^2^
*Whole− grain intake (g/10 MJ/d)*^*1*^* β (95% CI)*					
Body weight (kg) (n = 7006)					
Multivariate adjustment^3^					
Total WG	Ref (n 2292)	− 1.82 (− 3.74, 0.09)	− 1.26 (− 3.15, 0.63)	− 2.72 (− 4.12, − 1.31)	0.003
UPF WG^4^	Ref (n 2645)	− 1.11 (− 3.24, 1.02)	− 2.59 (− 4.42, − 0.76)	− 0.95 (− 2.47, 0.58)	0.20
Non-UPF WG^4^	Ref (n 5836)	− 2.51 (− 5.95, 0.93)	− 3.64 (− 6.43, − 0.84)	− 2.43 (− 4.89, 0.04)	0.002
RG adjustment^3,5^					
Total WG	Ref	− 1.82 (− 3.73, 0.09)	− 1.23 (− 3.13, 0.67)	− 2.64 (− 4.17, − 1.12)	0.008
UPF WG^4^	Ref	− 1.11 (− 3.23, 1.02)	− 2.58 (− 4.41, − 0.74)	− 0.9 (− 2.44, 0.64)	0.22
Non-UPF WG^4^	Ref	− 2.5 (− 5.94, 0.93)	− 3.59 (− 6.47, − 0.72)	− 2.33 (− 4.9, 0.23)	0.004
BMI (kg/m^2^) (n = 7003)					
Multivariate adjustment^3^					
Total WG	Ref (n 2292)	− 0.88 (− 1.52, − 0.24)	− 0.82 (− 1.47, − 0.18)	− 1.28 (− 1.81, − 0.76)	0.0002
UPF WG^4^	Ref (n 2645)	− 0.47 (− 1.22, 0.28)	− 1.12 (− 1.74, − 0.5)	− 0.64 (− 1.18, − 0.1)	0.03
Non-UPF WG^4^	Ref (n 5833)	− 1.16 (− 2.18, − 0.14)	− 1.43 (− 2.3, − 0.57)	− 1.16 (− 1.96, − 0.36)	< 0.0001
RG adjustment^3,5^					
Total WG	Ref	− 0.88 (− 1.51, − 0.24)	− 0.78 (− 1.43, − 0.14)	− 1.16 (− 1.71, − 0.61)	0.001
UPF WG^4^	Ref	− 0.47 (− 1.21, 0.27)	− 1.1 (− 1.71, − 0.48)	− 0.56 (− 1.11, − 0.02)	0.0499
Non-UPF WG^4^	Ref	− 1.15 (− 2.17, − 0.12)	− 1.37 (− 2.26, − 0.47)	− 1.0 (− 1.81, − 0.18)	0.0003
Waist circumference (cm) (n = 6847)					
Multivariate adjustment^3^					
Total WG	Ref (n 2243)	− 1.59 (− 3.21, 0.03)	− 1.25 (− 2.69, 0.2)	− 2.66 (− 3.97, − 1.36)	0.002
UPF WG^4^	Ref (n 2588)	− 0.69 (− 2.63, 1.26)	− 2.26 (− 3.68, − 0.84)	− 1.02 (− 2.32, 0.3)	0.11
Non-UPF WG^4^	Ref (n 5697)	− 2.71 (− 5.33, − 0.08)	− 3.18 (− 4.91, − 1.44)	− 2.54 (− 4.85, − 0.24)	0.001
RG adjustment^3,5^					
Total WG	Ref	− 1.58 (− 3.18, 0.02)	− 1.16 (− 2.6, 0.29)	− 2.4 (− 3.73, − 1.07)	0.01
UPF WG^4^	Ref	− 0.69 (− 2.6, 1.23)	− 2.21 (− 3.61, − 0.8)	− 0.83 (− 2.15, 0.48)	0.17
Non-UPF WG^4^	Ref	− 2.69 (− 5.31, − 0.06)	− 3.03 (− 4.81, − 1.25)	− 2.2 (− 4.48, 0.09)	0.001
Waist- to-height ratio (n = 6843)					
Multivariate adjustment^3^					
Total WG	Ref (n 2241)	− 0.01 (− 0.02, − 0.001)	− 0.01 (− 0.02, − 0.003)	− 0.02 (− 0.03, − 0.01)	0.001
UPF WG^4^	Ref (n 2586)	− 0.005 (− 0.02, 0.008)	− 0.02 (− 0.02, − 0.007)	− 0.009 (− 0.02, − 0.001)	0.03
Non-UPF WG^4^	Ref (n 5693)	− 0.02 (− 0.03, − 0.005)	− 0.02 (− 0.03, − 0.01)	− 0.02 (− 0.03, − 0.004)	0.0001
RG adjustment^3,5^					
Total WG	Ref	− 0.01 (− 0.02, − 0.001)	− 0.01 (− 0.02, − 0.002)	− 0.02 (− 0.02, − 0.008)	0.003
UPF WG^4^	Ref	− 0.005 (− 0.02, 0.008)	− 0.02 (− 0.02, − 0.007)	− 0.007 (− 0.02, 0.0008)	0.07
Non-UPF WG^4^	Ref	− 0.02 (− 0.03, − 0.005)	− 0.02 (− 0.03, − 0.009)	− 0.01 (− 0.03, − 0.001)	0.0005
Systolic blood pressure (mmHg) (n = 6752)					
Multivariate adjustment^5^					
Total WG	Ref (n 2207)	− 1.16 (− 2.82, 0.5)	− 0.48 (− 2.11, 1.16)	− 0.97 (− 2.53, 0.58)	0.35
UPF WG^4^	Ref (n 2546)	− 0.74 (− 2.31, 0.83)	− 0.91 (− 2.57, 0.74)	− 0.32 (− 2.06, 1.42)	0.78
Non-UPF WG^4^	Ref (n 5614)	− 0.73 (− 2.86, 1.4)	− 1.51 (− 3.83, 0.81)	1.43 (− 1.76, 4.63)	0.78
RG adjustment^3,5^					
Total WG	Ref	− 1.15 (2.81, 0.51)	− 0.42 (− 2.06, 1.22)	− 0.79 (− 2.33, 0.75)	0.50
UPF WG^4^	Ref	− 0.73 (− 2.31, 0.85)	− 0.87 (− 2.52, 0.79)	− 0.17 (− 1.88, 1.54)	0.92
Non-UPF WG^4^	Ref	− 0.72 (− 2.83, 1.4)	− 1.38 (− 3.72, 0.96)	1.72 (− 1.43, 4.87)	0.61
BMI adjustment^6,7^					
Total WG	Ref	− 0.8 (− 2.45, 0.85)	− 0.18 (− 1.79, 1.43)	− 0.44 (− 1.95, 1.07)	0.77
UPF WG^4^	Ref	− 0.53 (− 2.05, 1.0)	− 0.48 (− 2.18, 1.22)	− 0.07 (− 1.73, 1.59)	0.99
Non-UPF WG^4^	Ref	− 0.23 (− 2.38, 1.92)	− 0.94 (− 3.17, 1.29)	1.88 (− 1.3, 5.05)	0.43
WC adjustment^6,8^					
Total WG	Ref	− 0.88 (− 2.54, 0.78)	− 0.26 (− 1.85, 1.33)	− 0.53 (− 2.06, 1.0)	0.73
UPF WG^4^	Ref	− 0.6 (− 2.15, 0.95)	− 0.52 (− 2.21, 1.17)	− 0.17 (− 1.82, 1.49)	0.93
Non-UPF WG^4^	Ref	− 0.24 (− 2.4, 1.92)	− 0.96 (− 3.2, 1.28)	1.85 (− 1.27, 4.98)	0.42
Diastolic blood pressure (mmHg) (n = 6734)					
Multivariate adjustment^5^					
Total WG	Ref (n 2203)	− 0.32 (− 1.45, 0.81)	− 0.94 (− 1.85, − 0.03)	− 1.23 (− 2.15, − 0.31)	0.03
UPF WG^4^	Ref (n 2542)	− 0.45 (− 1.73, 0.84)	− 0.25 (− 1.18, 0.69)	− 1.27 (− 2.22, − 0.32)	0.03
Non-UPF WG^4^	Ref (n 5599)	− 0.93 (− 2.49, 0.63)	− 1.19 (− 3.13, 0.76)	− 0.0002 (− 1.31, 1.31)	0.32
RG adjustment^3,5^					
Total WG	Ref	− 0.31 (− 1.43, 0.81)	− 0.9 (− 1.78, − 0.03)	− 1.11 (− 2.0, − 0.21)	0.04
UPF WG^4^	Ref	− 0.45 (− 1.72, 0.83)	− 0.22 (− 1.13, 0.69)	− 1.18 (− 2.11, − 0.25)	0.04
Non-UPF WG^4^	Ref	− 0.93 (− 2.48, 0.63)	− 1.11 (− 3.08, 0.86)	0.17 (− 1.11, 1.45)	0.44
BMI adjustment^6,7^					
Total WG	Ref	− 0.13 (− 1.22, 0.97)	− 0.79 (− 1.69, 0.11)	− 0.95 (− 1.86, − 0.04)	0.08
UPF WG^4^	Ref	− 0.34 (− 1.61, 0.93)	− 0.02 (− 0.93, 0.89)	− 1.14 (− 2.08, − 0.2)	0.04
Non-UPF WG^4^	Ref	− 0.67 (− 2.14, 0.8)	− 0.89 (− 2.81, 1.03)	0.24 (− 1.07, 1.55)	0.58
WC adjustment^6,8^					
Total WG	Ref	− 0.16 (− 1.27, 0.95)	− 0.82 (− 1.72, 0.08)	− 0.98 (− 1.9, − 0.06)	0.06
UPF WG^4^	Ref	− 0.37 (− 1.66, 0.92)	− 0.02 (− 0.93, 0.89)	− 1.19 (− 2.13, − 0.24)	0.04
Non-UPF WG^4^	Ref	− 0.65 (− 2.11, 0.8)	− 0.88 (− 2.77, 1.02)	0.24 (− 1.07, 1.56)	0.59
Total cholesterol (mmol/L) (n = 5162)					
Multivariate adjustment^9^					
Total WG	Ref (n 1764)	0.007 (− 0.14, 0.15)	− 0.04 (− 0.14, 0.07)	− 0.01 (− 0.16, 0.13)	0.86
UPF WG^4^	Ref (n 2018)	0.009 (− 0.13, 0.15)	− 0.02 (− 0.12, 0.08)	0.02 (− 0.11, 0.16)	0.57
Non-UPF WG^4^	Ref (n 4319)	− 0.1 (− 0.31, 0.12)	0.07 (− 0.14, 0.28)	− 0.04 (− 0.2, 0.11)	0.10
RG adjustment^3,5^					
Total WG	Ref	0.006 (− 0.14, 0.15)	− 0.04 (− 0.14, 0.07)	− 0.02 (− 0.17, 0.13)	0.77
UPF WG^4^	Ref	0.009 (− 0.13, 0.15)	− 0.02 (− 0.12, 0.07)	0.01 (− 0.12, 0.15)	0.64
Non-UPF WG^4^	Ref	− 0.1 (− 0.31, 0.12)	0.06 (− 0.14, 0.27)	− 0.05 (− 0.21, 0.1)	0.07
BMI adjustment^7,9^					
Total WG	Ref	0.01 (− 0.13, 0.16)	− 0.03 (− 0.13, 0.07)	− 0.006 (− 0.15, 0.14)	0.94
UPF WG^4^	Ref	0.01 (− 0.12, 0.15)	− 0.01 (− 0.11, 0.08)	0.02 (− 0.11, 0.16)	0.56
Non-UPF WG^4^	Ref	− 0.09 (− 0.3, 0.12)	0.08 (− 0.13, 0.29)	− 0.04 (− 0.19, 0.12)	0.16
WC adjustment^8,9^					
Total WG	Ref	0.01 (− 0.13, 0.16)	− 0.03 (− 0.13, 0.08)	− 0.007 (− 0.15, 0.14)	0.82
UPF WG^4^	Ref	0.01 (− 0.12, 0.15)	− 0.01 (− 0.11, 0.08)	0.02 (− 0.11, 0.16)	0.56
Non-UPF WG^4^	Ref	− 0.08 (− 0.29, 0.12)	0.08 (− 0.13, 0.29)	− 0.03 (− 0.19, 0.12)	0.18
Fasting LDL-cholesterol (mmol/L) (n = 2324)					
Multivariate adjustment^9^					
Total WG	Ref (n 808)	0.008 (− 0.19, 0.2)	0.005 (− 0.13, 0.14)	− 0.01 (− 0.19, 0.17)	0.95
UPF WG^4^	Ref (n 917)	− 0.006 (− 0.2, 0.18)	− 0.04 (− 0.2, 0.12)	− 0.06 (− 0.23, 0.11)	0.54
Non-UPF WG^4^	Ref (n 1961)	0.002 (− 0.38, 0.38)	0.35 (0.04, 0.65)	− 0.005 (− 0.32, 0.31)	0.95
RG adjustment^3,5^					
Total WG	Ref	0.008 (− 0.19, 0.2)	0.003 (− 0.14, 0.14)	− 0.02 (− 0.2, 0.17)	0.95
UPF WG^4^	Ref	− 0.006 (− 0.2, 0.18)	− 0.04 (− 0.2, 0.12)	− 0.06 (− 0.24, 0.11)	0.53
Non-UPF WG^4^	Ref	0.003 (− 0.38, 0.38)	0.34 (0.04, 0.65)	− 0.009 (− 0.34, 0.32)	0.95
BMI adjustment^7,9^					
Total WG	Ref	0.03 (− 0.17, 0.22)	0.02 (− 0.12, 0.16)	− 0.005 (− 0.19, 0.18)	0.9993
UPF WG^4^	Ref	0.006 (− 0.19, 0.2)	− 0.02 (− 0.18, 0.14)	− 0.06 (− 0.23, 0.11)	0.58
Non-UPF WG^4^	Ref	0.04 (− 0.33, 0.4)	0.37 (0.07, 0.67)	0.01 (− 0.3, 0.31)	0.90
WC adjustment^8,9^					
Total WG	Ref	0.03 (− 0.16, 0.22)	0.02 (− 0.12, 0.16)	− 0.006 (− 0.19, 0.18)	0.86
UPF WG^4^	Ref	0.001 (− 0.19, 0.19)	− 0.02 (− 0.18, 0.14)	− 0.06 (− 0.24, 0.11)	0.57
Non-UPF WG^4^	Ref	0.05 (− 0.31, 0.41)	0.37 (0.07, 0.67)	0.02 (− 0.28, 0.32)	0.86
HDL-cholesterol (mmol/L) (n = 6559)					
Multivariate adjustment^9^					
Total WG	Ref (n 2157)	0.01 (− 0.03, 0.05)	0.05 (0.007, 0.09)	0.05 (0.006, 0.08)	0.02
UPF WG^4^	Ref (n 2487)	0.01 (− 0.02, 0.04)	0.05 (0.005, 0.09)	0.01 (− 0.03, 0.06)	0.48
Non-UPF WG^4^	Ref (n 5461)	0.04 (− 0.02, 0.1)	0.02 (− 0.03, 0.08)	0.04 (− 0.01, 0.1)	0.06
RG adjustment^3,5^					
Total WG	Ref	0.01 (− 0.03, 0.05)	0.04 (0.003, 0.08)	0.02 (− 0.01, 0.06)	0.19
UPF WG^4^	Ref	0.01 (− 0.02, 0.04)	0.04 (0.0009, 0.09)	− 0.002 (− 0.05, 0.04)	0.96
Non-UPF WG^4^	Ref	0.04 (− 0.02, 0.1)	0.01 (− 0.04, 0.07)	0.02 (− 0.04, 0.08)	0.31
BMI adjustment^7,9^					
Total WG	Ref	− 0.004 (− 0.04, 0.03)	0.04 (− 0.0008, 0.07)	0.02 (− 0.009, 0.05)	0.10
UPF WG^4^	Ref	0.002 (_0.03, 0.03)	0.03 (− 0.008, 0.07)	0.003 (− 0.04, 0.05)	0.73
Non-UPF WG^4^	Ref	0.02 (− 0.04, 0.07)	− 0.003 (− 0.05, 0.05)	0.03 (− 0.03, 0.08)	0.33
WC adjustment^8,9^					
Total WG	Ref	− 0.001 (− 0.04, 0.03)	0.04 (0.003, 0.07)	0.03 (− 0.005, 0.06)	0.08
UPF WG^4^	Ref	0.005 (− 0.03, 0.04)	0.03 (− 0.007, 0.07)	0.008 (− 0.03, 0.05)	0.68
Non-UPF WG^4^	Ref	0.01 (− 0.04, 0.06)	− 0.003 (− 0.06, 0.05)	0.03 (− 0.03, 0.08)	0.29
Fasting triglycerides (mmol/L)^10^ (n = 2233)					
Multivariate adjustment^11^					
Total WG	Ref (n 776)	0.97 (0.9, 1.05)	0.98 (0.9, 1.0)	1.03 (0.93, 1.14)	0.92
UPF WG^4^	Ref (n 881)	1.05 (0.94, 1.16)	1.01 (0.91, 1.13)	1.06 (0.98, 1.15)	0.48
Non-UPF WG^4^	Ref (n 1885)	0.86 (0.67, 1.09)	1.04 (0.91, 1.19)	0.87 (0.73, 1.04)	0.24
RG adjustment^3,5^					
Total WG	Ref	0.97 (0.89, 1.05)	0.99 (0.9, 1.08)	1.05 (0.95, 1.17)	0.48
UPF WG^4^	Ref	1.05 (0.94, 1.16)	1.02 (0.91, 1.14)	1.08 (1.0, 1.17)	0.27
Non-UPF WG^4^	Ref	0.85 (0.67, 1.08)	1.05 (0.92, 1.19)	0.89 (0.75, 1.05)	0.36
BMI adjustment^7,11^					
Total WG	Ref	1.0 (0.93, 1.08)	1.0 (0.92, 1.09)	1.04 (0.94, 1.15)	0.83
UPF WG^4^	Ref	1.06 (0.96, 1.18)	1.04 (0.94, 1.16)	1.06 (0.99, 1.14)	0.42
Non-UPF WG^4^	Ref	0.9 (0.72, 1.11)	1.07 (0.94, 1.22)	0.89 (0.75, 1.04)	0.42
WC adjustment^8,11^					
Total WG	Ref	1.0 (0.92, 1.08)	1.0 (0.91, 1.09)	1.04 (0.94, 1.15)	0.83
UPF WG^4^	Ref	1.05 (0.95, 1.17)	1.04 (0.94, 1.15)	1.66 (0.99, 1.14)	0.42
Non-UPF WG^4^	Ref	0.91 (0.74, 1.13)	1.07 (0.94, 1.22)	0.89 (0.76, 1.04)	0.50
Fasting plasma glucose (mmol/L)^10^ (n = 3040)					
Multivariate adjustment^12^					
Total WG	Ref (n 1019)	1.0 (0.97, 1.02)	1.0 (0.97, 1.03)	1.02 (0.99, 1.06)	0.20
UPF WG^4^	Ref (n 1162)	1.01 (0.98, 1.04)	1.0 (0.97, 1.03)	1.02 (0.98, 1.06)	0.33
Non-UPF WG^4^	Ref (n 2560)	0.96 (0.93, 0.99)	0.97 (0.93, 1.0)	1.03 (0.96, 1.11)	0.81
RG adjustment^3,5^					
Total WG	Ref	1.0 (0.97, 1.02)	1.0 (0.97, 1.03)	1.03 (0.99, 1.07)	0.16
UPF WG^4^	Ref	1.01 (0.98, 1.04)	1.0 (0.97, 1.03)	1.02 (0.99, 1.06)	0.28
Non-UPF WG^4^	Ref	0.96 (0.93, 0.99)	0.97 (0.93, 1.01)	1.03 (0.96, 1.11)	0.76
BMI adjustment^7,12^					
Total WG	Ref	1.0 (0.98, 1.02)	1.01 (0.98, 1.04)	1.03 (0.99, 1.07)	0.12
UPF WG^4^	Ref	1.01 (0.98, 1.04)	1.01 (0.98, 1.04)	1.02 (0.99, 1.06)	0.28
Non-UPF WG^4^	Ref	0.97 (0.94, 1.01)	0.98 (0.95, 1.01)	1.04 (0.96, 1.12)	0.57
WC adjustment^8,12^					
Total WG	Ref	1.0 (0.98, 1.02)	1.01 (0.98, 1.04)	1.03 (0.99, 1.07)	0.10
UPF WG^4^	Ref	1.01 (0.98, 1.04)	1.01 (0.98, 1.04)	1.02 (0.98, 1.06)	0.24
Non-UPF WG^4^	Ref	0.98 (0.95, 1.01)	0.98 (0.95, 1.01)	1.04 (0.96, 1.12)	0.51
HbA1c (%)^10^ (n = 6283)					
Multivariate adjustment^12^					
Total WG	Ref (n 2061)	1.0 (0.99, 1.01)	1.01 (0.99, 1.02)	1.0 (0.99, 1.02)	0.77
UPF WG^4^	Ref (n 2378)	1.0 (0.99, 1.01)	1.0 (0.99, 1.01)	1.01 (0.99, 1.03)	0.15
Non-UPF WG^4^	Ref (n 5234)	0.99 (0.97, 1.0)	1.0 (0.97, 1.03)	0.99 (0.97, 1.01)	0.32
RG adjustment^3,5^					
Total WG	Ref	1.0 (0.99, 1.01)	1.01 (0.99, 1.02)	1.0 (0.99, 1.02)	0.56
UPF WG^4^	Ref	1.0 (0.99, 1.01)	1.0 (0.99, 1.01)	1.01 (1.0, 1.03)	0.11
Non-UPF WG^4^	Ref	0.99 (0.97, 1.0)	1.0 (0.97, 1.03)	0.99 (0.98, 1.01)	0.45
BMI adjustment^7,12^					
Total WG	Ref	1.0 (0.99, 1.01)	1.01 (1.0, 1.02)	1.01 (0.99, 1.02)	0.43
UPF WG^4^	Ref	1.0 (0.99, 1.01)	1.0 (0.99, 1.01)	1.01 (1.0, 1.03)	0.10
Non-UPF WG^4^	Ref	0.99 (0.98, 1.01)	1.0 (0.98, 1.04)	0.99 (0.98, 1.01)	0.74
WC adjustment^8,12^					
Total WG	Ref	1.0 (0.99, 1.01)	1.01 (1.0, 1.02)	1.0 (0.99, 1.02)	0.51
UPF WG^4^	Ref	1.0 (0.99, 1.01)	1.0 (0.99, 1.01)	1.01 (1.0, 1.03)	0.12
Non-UPF WG^4^	Ref	0.99 (0.98, 1.01)	1.01 (0.98, 1.04)	0.99 (0.98, 1.01)	0.73
C-reactive protein (CRP)^10^ (mg/L) (n = 5139)					
Multivariate adjustment^9^					
Total WG	Ref (n 1759)	0.92 (0.81, 1.05)	0.81 (0.7, 0.94)	0.81 (0.71, 0.91)	< 0.0001
UPF WG^4^	Ref (n 2012)	0.97 (0.84, 1.12)	0.85 (0.74, 0.98)	0.9 (0.77, 1.04)	0.02
Non-UPF WG^4^	Ref (n 4296)	0.77 (0.6, 0.98)	0.81 (0.69, 0.95)	0.81 (0.65, 1.0)	0.0003
RG adjustment^3,5^					
Total WG	Ref	0.92 (0.81, 1.05)	0.8 (0.7, 0.93)	0.79 (0.7, 0.89)	< 0.0001
UPF WG^4^	Ref	0.97 (0.84, 1.12)	0.85 (0.74, 0.97)	0.88 (0.75, 1.03)	0.013
Non-UPF WG^4^	Ref	0.77 (0.6, 0.97)	0.8 (0.68, 0.94)	0.78 (0.64, 0.96)	< 0.0001
BMI adjustment^7,9^					
Total WG	Ref	1.0 (0.9, 1.11)	0.87 (0.76, 1.0)	0.88 (0.78, 0.98)	0.0003
UPF WG^4^	Ref	1.03 (0.92, 1.15)	0.93 (0.82, 1.05)	0.94 (0.82, 1.08)	0.05
Non-UPF WG^4^	Ref	0.84 (0.7, 1.01)	0.91 (0.8, 1.04)	0.89 (0.72, 1.1)	0.04
WC adjustment^8,9^					
Total WG	Ref	0.99 (0.9, 1.09)	0.87 (0.76, 0.99)	0.86 (0.76, 0.97)	0.0001
UPF WG^4^	Ref	1.01 (0.91, 1.12)	0.92 (0.81, 1.04)	0.92 (0.8, 1.05)	0.02
Non-UPF WG^4^	Ref	0.84 (0.69, 1.02)	0.92 (0.8, 1.05)	0.88 (0.71, 1.09)	0.03

BMI, body mass index; non- UPF, non-ultra-processed food; RG,refined grain; UPF,ultra-processed food; WC,waist circumference; WG,whole grain

^1^Values reported as β coefficient (95% CI)

^2^*P*-value for linear trend. A significance is determined at *p* < 0.05

^3^Survey linear regression adjusted for age, sex, education, physical activity, smoking status, energy intake, alcohol intake, PIR, ethnicity, and country of birth

^4^Simultaneously adjusted for non- ultra-processed and ultra- processed whole-grain intake

^5^Multivariate adjusted linear regression adjusted for refined grain intake

^6^Survey linear regression adjusted for age, sex, education, physical activity, smoking status, energy intake, alcohol intake, PIR, ethnicity, country of birth, and Na intake

^7^Survey linear regression adjusted for BMI

^8^Survey linear regression adjusted for WC

^9^Survey linear regression adjusted for age, sex, education, physical activity, smoking status, energy intake, alcohol intake, PIR, ethnicity, country of birth, saturated fat intake, monounsaturated fat intake, and polyunsaturated fat intake

^10^Reported as exp(β) of the natural log transformed variable

^11^Survey linear regression adjusted for age, sex, education, physical activity, smoking status, energy intake, alcohol intake, PIR, ethnicity, country of birth, saturated fat intake, monounsaturated fat intake, polyunsaturated fat intake, and added sugar intake

^12^Survey linear regression adjusted for age, sex, education, physical activity, smoking status, energy intake, alcohol intake, PIR, ethnicity, country of birth, and added sugar intake

Overall results were slightly different between Australia and the US, but the trends of associations were similar. Particularly in the UPF comparisons there appeared a U-shaped relationship where the highest tertile was not exhibiting the greatest effect, although the lack of significance in nearly all the comparisons limits conclusions. In Australia, no statistically significant associations were observed between any categories of whole-grain intake (total, UPF, and non-UPF) and SBP, cholesterol (total, LDL, and HDL), fasting triglycerides, and HbA1c. Similarly, there were no statistically significant associations between total whole-grain intake and body weight, WC, and CRP; UPF whole-grain intake and body weight, BMI, fasting plasma glucose, and CRP; and non-UPF whole-grain intake and DBP. In the US, non-significant associations were observed between any categories of whole-grain intake (total, UPF, and non-UPF) and SBP, total and LDL cholesterol, fasting triglycerides, fasting plasma glucose, and HbA1c. There were only non-significant associations between UPF whole-grain intake and body weight, WC, and HDL cholesterol, and non-UPF and DBP. 

#### Anthropometric

Generally, significant inverse associations were found between anthropometric measures and whole grain intake **(**Table 3 and 4**).** In particular, all categories (total, UPF, and non-UPF) were associated with lower BMI in the US, and in both the US and Australia after adjusting for refined grain intake (in non-adjusted for refined grain Australian data, the association was significant only for total and non-UPF whole grains). Similarly, significant lower WHR was noted for all categories of whole grain intake in both countries (excluding refined grain adjusted UPF whole-grain intake in the US). Significant inverse associations were found between total and non-UPF whole-grain intake and body weight in the US (*p* = 0.003 and *p* = 0.002, respectively), and for non-UPF whole-grain intake only in Australia (*p* = 0.02). These associations remained significant when adjusted for refined grain intake, and in Australia for body weight, a significant inverse association was found for UPF whole-grain intake (*p* = 0.03) following refined grain adjustment.

In Australia, significant inverse associations were found between non-UPF and UPF whole grains and WC (*p* = 0.02 and *p* = 0.0496, respectively). This remained when adjusted for refined grain intake and was also found for total whole-grain intake (*p* = 0.02). In the US, significant inverse associations were found for total and non-UPF whole grains regarding WC (*p* = 0.002 and *p* = 0.001, respectively), including when adjusted for refined grain intake.

#### Blood pressure

Significant inverse associations were found between total and UPF whole-grain intake and DBP in Australian (*p* = 0.007 and *p* = 0.005, respectively) and US adults (*p* = 0.04 and *p* = 0.001, respectively). In Australia and the US, the inverse associations weakened slightly after additional adjustments of BMI and WC although remained statistically significant, except that the associations for total whole grains in the US became statistically insignificant after additional adjustments of BMI and WC.

#### Lipids

In the US, higher total whole-grain intake was associated with higher HDL-cholesterol (*p* = 0.02) but this association no longer statistically significant after adjustment for refined grains, BMI, and WC.

#### Glucose metabolism

Higher total and non-UPF whole-grain intake were associated with lower fasting plasma glucose in Australia (*p* = 0.046 and *p* = 0.002, respectively). Only non-UPF whole-grain intake remained significant when adjusted for refined grain intake, BMI, and WC.

#### CRP

Significant inverse associations were found between non-UPF whole grains and CRP in Australia, independent of refined grains (*p* = 0.02), however associations were attenuated after adjustment for WC and BMI. In the US, all categories of whole-grain intake were inversely associated with CRP (total *p* < 0.0001; UPF *p* = 0.02; non-UPF = *p* 0.0003). This did not remain significant for UPF whole grains when adjusted for BMI.

## Discussion

This analysis is a nationally representative cross-sectional study exploring intakes of whole grain from sources considered non-UPF versus UPF by the Nova food classification system in both Australian and US adult populations and related associations with cardiometabolic risk measures. This work highlights the nuances of food processing in relation to nutrition and health outcomes, particularly for whole grains, that are accepted as beneficial for health but often require processing for safety, palatability, and convenience. The current analysis identifies that whole-grain foods of all levels of processing remain beneficial for health however it is possible the benefit on risk factors may be maximised where processing is limited. Choosing less processed whole grains, however, may involve reduced convenience, including longer preparation times and the need for kitchen access and sufficient cooking skills.

In this study, while many associations for cardiometabolic risk measures were not significant, interesting results were noted for anthropometric outcomes. Significant inverse associations were found between intakes of non-UPF whole grains and body weight, BMI, WC, and WHR in Australia and the US. Significant inverse associations were still found between intakes of UPF whole grains and WHR and BMI in the US; however, the magnitude of effect when comparing tertiles was lesser compared to the magnitude of the effect of non-UPF whole grains. Given the health protective effects of UPF whole grains observed in the current study and previous research [11–13], foods may be better represented as a continuum within the Nova UPF category to address where nuance in health associations exist related to nutrient compositions.

The associations between whole-grain intake and health benefits observed in this study are generally weaker than those reported in previous research [36]. This is likely due, at least in part, to the low whole-grain intake especially in the US population, which becomes even lower when intake is disaggregated by Nova categories. Low median intakes of whole grains across all categories indicate a significant proportion of both populations consume no whole grains and therefore both populations do not meet recommended intake target of 48 g/d (or otherwise described as 90 g or three 1-oz servings of whole-grain food) like many countries globally [18]. The focus of whole-grain promotional efforts should be placed on improving overall population whole-grain intakes as the priority over specific messaging related to processing. In any consideration of promotional efforts to prioritise non-UPF sources, an understanding of available food supplies is required. It would be difficult to increase global intakes of whole grains while excluding UPF varieties entirely as this study found UPF whole grains comprise more than two-thirds of total intakes in the US and half of intake in Australia. In particular, to improve access to non-UPF whole-grain products, food manufactures may need to be incentivised to develop and market whole-grain products of limited processing.

Non-UPF whole-grain intake in Australia was also inversely associated with fasting plasma glucose. This may relate to the degree of grain milling as interventional evidence has shown that consuming less processed whole-grain foods improves glucose related measures compared with similar amounts of more finely milled grain foods among individuals with T2D [37]. Conversely, other research suggests that finer milling of whole-grain foods enhances fermentation and phytase activity, thereby improving nutrient bioavailability and offers metabolic benefits [38, 39]. In Australia, it is likely that a substantial contributor to non-UPF whole-grain intake is oats and oatmeal. This may also explain the protective association with fasting plasma glucose for non-UPF whole-grain intake only as previous trials throughout the literature have closely analysed the strong link between intake of oats and favourable fasting plasma glucose levels [40]. It is also understood that consumption of oats has less of an indication for reductions in HbA1c even alongside reductions in fasting plasma glucose [40], as also apparent in the current study.

Only UPF whole-grain intake was inversely associated with DBP, and non-UPF whole-grain intake was inversely associated with CRP, in both Australia and the US (remaining significant at the tertile level). Trends for most counterpart categories (non-UPF whole grain and DBP; UPF whole grain and CRP) were in the same direction but were not statistically significant and the inconsistency may be related to statistical limitations of the small sample size. For DBP, this finding could also be influenced by the use of blood pressure lowering medication as data to exclude such participants were not available in Australia. Nevertheless, the result is consistent with previous interventional research showing that DBP improved with consumption of commercially available whole-grain foods compared with refined grains, while most other measures remained unchanged [41].

Adjustment of refined grain intake generally resulted in stronger associations across cardiometabolic risk measures for all categories of whole grains in Australia. However, the adjusting for refined grains attenuated associations between whole grains and cardiometabolic risk measures in the US. Observational research has indicated an inverse or null association between refined grain intake and mortality, risk of heart disease, blood pressure, stroke, T2D, cancer, or obesity [42]. This suggests some refined grain consumption is, at least, not deleterious. Participants in the highest tertile of whole-grain intake in Australia consumed higher amounts of whole grain (83.0 g/10 MJ/day) compared to those in the US in the same tertile of intake (61.4 g/10 MJ/day) which may explain the stronger associations found after adjustment, particularly given relatively similar sample sizes (Supplementary Material 5 and 6). Additionally, refined grain intake in the highest tertile in the US (80.0 g/10 MJ/day) was consumed more in excess relative to whole-grain intake in the same tertile when compared to Australia (92.3 g/10 MJ/day) which may in part explain attenuated associations found.

Various mechanisms of actions have been proposed as to how UPF negatively impact health [8]. One proposed trait of UPF that harms health is hyper-palatability, in part caused by disruption to the food matrix, leading to increased energy intake [43, 44]. The palatability of foods has been shown to impact satiation responses when eating [45] and grains more broadly are considered hyperpalatable [40]. However, differences exist between satiety response of whole wheat versus refined varieties of bread, for example, where the satiety onset of whole wheat is stronger compared to refined [46]. Given whole-grain containing UPF are not consumed in excess in both populations in the current study and previous research [14, 15], this proposed mechanism may not extend to the whole-grain UPF subgroup. Further research investigating mechanisms of actions directly comparing non-UPF and UPF is needed, particularly for whole grains were nuanced evidence exists.

While the promotion of less processed food sources needs to be a public health priority, it is still important to consider nutrient compositions and broader dietary patterns [11, 12]. For example, in the US the added sugar content of UPF has previously been flagged as a concern for poor health [47]. However, based on the nutrient analyses in this study (Supplementary Material 6), no significant associations were found for UPF whole-grain consumers and added sugar intake in US adults. This may be due to correlated intakes of other foods as people who consume whole grains including ultra-processed varieties may seek healthier diets. This nuance should be considered when comparing to studies that have found direct association between intakes of the broader UPF category and added sugars [48]. Messaging should move towards encompassing all aspects of food including nutritional density and food groups as well as including concepts such as processing and food environments to attempt to address these nuances and thereby support population understanding and improved overall dietary health impacts.

This study is not without limitations. The NNPAS 2011-12 is the most recent nationally representative data available in Australia, however given that it is greater than 10 years old, current intakes in Australia may be different to what is reported in the current study and is therefore limited by this. NHANES 2015-18 provides the most up-to-date dietary intake data for the US, pre-Covid. The cross-sectional study design here also only infers correlation between exposure and outcomes, and cannot comment on causation, including reverse causation for significant associations, for both populations. Beneficial cross-sectional associations between whole-grain intake and anthropometric outcomes and health behaviours, however, are well documented throughout literature. Adjustments for multiple outcomes and multiple comparisons were performed on characteristic analyses and not primary analyses in this study and should be considered when interpreting the findings. The similar dietary data collection methods employed in NNPAS 2011-12 and NHANES 2016-18, typical of most nationally representative surveys, are limited to intake estimation and may not reflect true intakes. To reduce the effects of this limitation, this study has used an average of two days of dietary intake data for both populations, however it is recommended in literature to use at least four 24-h recalls to accurately capture usual intakes and ensure rigorous reporting [49]. To control for misreporting bias inherent in 24-h recall methods, like the tendency to underreport intakes due to social desirability, 99.7% confidence interval eligibility criteria was applied in the current study. The conversion of whole grain data from ounce equivalent measures to grams per day in the US may result in less precise estimates than the directly reported gram-based data in Australia, potentially leading to discrepancies in the results. Findings in this study may be confounded by correlated dietary patterns (for example, people who consume more UPF whole grains but not other types of UPF, may also tend to consume more of other health-protective foods including fruits and vegetables). Lastly, the researchers who developed both the AUSNUT 2011-13 Nova database and the NHANES Nova 2015-18 database have acknowledged variability in information provided on processing in both databases as they were not intended for this purpose [28, 29]. This is especially the case for the NHANES database where all non-homemade bread were classified as UPF as a result.

This study is strengthened by using nationally representative data of both the US and Australia that includes detailed dietary data collection methods, specifically 24-h diet recalls. The use of nationally representative data allowed application of survey weighting in study methods respective for both populations [21, 23]. This ensures that results from this study are generalisable to the Australian population and US population at the time of each survey. This study is also the first to distinguish whole-grain foods by their level of processing using the Nova classification system, as well as the first of its kind to consider the differences in cardiometabolic risk measures when consuming non-UPF versus UPF sources of whole-grain foods. The findings of this study contribute to the evidence and call for further research regarding the effects of level of processing.

This study found whole-grain intake is predominantly from ultra-processed sources in the US but consists of a relatively even mix of non-UPF and UPF sources in Australia, at the time of the survey. Higher intakes of both non-UPF and UPF whole-grain intake were associated with lower anthropometric, DBP, and CRP outcomes however the magnitude of the effect was stronger for non-UPF whole grains compared to UPF in these measures (other than DBP). All whole grain foods should be promoted in public health messaging and consumer guidance, but greater emphasis may be placed on less processed sources due to their stronger health benefits.

## Supplementary Information

Below is the link to the electronic supplementary material.

Supplementary file1 (DOCX 65 kb)

## Data Availability

Data described in the manuscript, code book, and analytic code will be made available upon request pending application and approval.

## Abbreviations

ABS: Australian Bureau of Statistics
ADG: Australian Dietary Guidelines
AUSNUT: Australian Food and Nutrient
AMPM: Automated multiple pass method
BMI: Body mass index
CVD: Cardiovascular disease
CRP: Centers for Disease Control and Prevention
CDC: C-reactive protein
DBP: Diastolic blood pressure
DGA: Dietary guidelines for americans
FNDDS: Food and nutrient database for dietary studies
FPED: Food patterns equivalents database
HbA1c: Glycated haemoglobin
HDL: High-density lipoprotein
IQR: Inter-quartile range
LDL: Low-density lipoprotein
MEC: Mobile examination centre
NHANES: National Health and Nutrition Examination Survey
NNPAS: National Nutrition and Physical Activity Survey
non-UPF: Non-ultra-processed food
SBP: Systolic blood pressure
T2D: Type 2 diabetes
UPF: Ultra-processed food
US: United States
WC: Waist circumference
WHR: Weight-to-height ratio
WGI: Whole grain initiative
24-h: 24-hour
