# Supplementary material for: Comparison of associations of intake of ultra-processed and non-ultra-processed whole-grain foods with cardiometabolic risk measures in Australian and US adults
Source: Eur J Nutr. 2026 Mar 17;65(3):93. doi: 10.1007/s00394-026-03942-8 (PMC12996004; doi:10.1007/s00394-026-03942-8)
Supplement: Supplementary file 1 — Supplementary file1 (DOCX 65 kb) [file 394_2026_3942_MOESM1_ESM.docx]

**Comparison of associations of whole-grain intake from ultra-processed versus non-ultra-processed foods with cardiometabolic risk factors in Australian and US adults.**

Elissa J. Price^1^, Mengxi Du^2,3,4^, Eden M. Barrett^1,5^, Nicola M. McKeown^6^, Marijka J. Batterham^1,7^, Fang Fang Zhang^8^, Eurídice Martínez Steele^9^, Eleanor J. Beck^1,7,*^

**Supplementary Material 1** Participant Flowchart NNPAS 2011-12

**Supplementary Material 2** Participant Flowchart NHANES 2015-18

**Supplementary Material 3** Description of covariates included in Australian characteristic and cardiometabolic risk measure regression analyses.

Covariates include:

- Age: participants age in years.
- Sex: male or female.
- Nutrient intakes: energy (kJ/d), fibre (g/d), sodium (mg/d), saturated fat intake (g/d), polyunsaturated fat intake (g/d), monounsaturated fat intake (g/d), trans fat intake (mg/d) and free sugar intake (% energy).
- Alcohol intake (g/day).
- Smoking status: current daily, current weekly (at least once weekly but not daily), current less than weekly, ex-smoker, or never smoked.
- Education level: postgraduate degree, graduate diploma, bachelor’s degree, advanced diploma, certificate iii/iv, certificate i/ii, undefined certificate, or no non-school qualification.
- Physical activity level: high, moderate, low, sedentary (very low), or sedentary (no exercise).
- Country of birth: Australia, or other.
- Remoteness area categories from the Australian Statistical Geography Standard (ASGS) 2016 [51]: major cities of Australia, inner regional Australia, or other.
- National Index of Relative Socio-economic Disadvantage 2011 (SEIFA) [52]: decile 1 – lowest to decile 10 – highest.

**Supplementary Material 4** Description of covariates included in US characteristic and cardiometabolic risk measure regression analyses.

Covariates include:

- Age: participants age in years.
- Sex: male or female.
- Nutrient intakes: energy (kJ/d), fibre (g/d), sodium (mg/d), saturated fat intake (g/d), polyunsaturated fat intake (g/d), monounsaturated fat intake (g/d), and added sugar intake (g/d).
- Alcohol consumption: non, moderate, or high consumption.
- Smoking status: current, previous, or non-smoker.
- Education level: <12 years education, 12 years education, and >12 years education.
- Physical activity status: yes or no.
- Metabolic equivalents of task (MET) score: MET score multiplied by minutes of moderate to vigorous work-related and leisure-time physical activity in a week, then summed to obtain a total estimate.
- Country of birth: born in the US, or other.
- Ethnicity: Mexican American, Other Hispanic, Non-Hispanic White, Non-Hispanic Black, or other Race including Multi-Racial.
- Ratio of family income to the US Bureau of Census poverty threshold (PIR) <1.3, 1.30-1.84, 1.85-2.99 ≥3).

**Supplementary Material 5** Population characteristics of non-consumers and consumers of whole grains by level of processing and in Australian adults (n 5102).

|  | **Tertiles of whole-grain intake (g/10MJ/d)** | | | | |
| --- | --- | --- | --- | --- | --- |
|  | **Non consumers** | **T1** | **T2** | **T3** | p-value^2^ |
| **Total whole-grain consumers^1^** | | | | | |
| n (unweighted) | 704 | 1434 | 1420 | 1544 | - |
| Median whole-grain intake (g/10MJ/day)(IQR) | 0 | 11.58 (4.17, 18.45) | 40.15 (33.45, 48.6) | 83.03 (69.91, 105.3) | - |
| Median non UPF whole-grain intake (g/10MJ/day)(IQR) | 0 | 0 (0, 9.08) | 18.04(0, 33.53) | 48.88 (21.33, 70.89) | - |
| Median UPF whole-grain intake (g/10MJ/day)(IQR) | 0 | 3.8 (0.71, 11.96) | 21.55(2.59, 34.37) | 38.22(10.45, 65.47) | - |
| Median refined grain intake (g/day)(IQR) | 122.5(76.55-172.6) | 124.0(92.16-163.4) | 103.0(70.89-144.4) | 92.3(60.11-128.9) | - |
| Age^3,4^ | 43.86(0.89)^AB^ | 43.47(0.48)^A^ | 46.5(0.6)^B^ | 51.09(0.62) | <0.0001 |
| Female (%)^5^ | 43.5(2.61)^A^ | 49.64(1.76)^A^ | 47.9(1.74)^A^ | 49.94(1.75)^A^ | 0.2239 |
| Energy intake (kJ/day)^3,6^ | 7715.1(132.7) | 9005.7(105.0)^B^ | 8704.4(84.0)^AB^ | 8411.9(93.7)^A^ | 0.0005 |
| Fibre intake (g/day)^3,7^ | 18.09(0.42) | 19.92(0.24) | 23.57(0.24) | 28.22(0.28) | <0.0001 |
| Sodium intake (mg/day)^3,7^ | 2513.8(45.01)^B^ | 2482.1(31.71)^B^ | 2353.8(28.32)^A^ | 2268.7(28.31)^A^ | <0.0001 |
| Saturated fat intake (g/day)^3,7^ | 28.98(0.52)^A^ | 28.52(0.3)^A^ | 27.45(0.32)^A^ | 25.28(0.26) | <0.0001 |
| Polyunsaturated fat intake (g/day)^3,7^ | 11.05(0.33)^AB^ | 11.13(0.2)^A^ | 11.5(0.22)^AB^ | 12.03(0.19)^B^ | 0.0062 |
| Monounsaturated fat intake (g/day)^3,7^ | 29.5(0.53)^A^ | 29.09(0.33)^A^ | 28.08(0.35)^A^ | 26.68(0.29) | <0.0001 |
| Trans fat intake (mg/day)^3,7^ | 1478.0(38.85)^B^ | 1422.4(24.32)^AB^ | 1339.3(22.61)^A^ | 1246.0(19.37) | <0.0001 |
| Free sugar intake (% energy)^3,6^ | 11.05(0.4)^A^ | 10.5(0.21)^A^ | 10.2(0.27)^A^ | 8.17(0.19) | <0.0001 |
| Alcohol intake (g/day)^3,7^ | 19.37(1.48)^B^ | 16.2(0.83)^AB^ | 13.61(0.79)^A^ | 9.45(0.7) | <0.0001 |
| University graduate (%)^5^ | 15.04(2.21)^A^ | 19.67(1.52)^AB^ | 21.25(1.68)^B^ | 19.15(1.54)^AB^ | 0.0114 |
| Current smoker daily (%)^5^ | 30.02(2.54) | 14.73(1.4)^A^ | 12.66(1.54)^A^ | 9.34(0.95)^A^ | <0.0001 |
| Low physical activity level (%)^5^ | 36.05(2.25) | 37.79(1.69)^A^ | 33.12(1.84)^A^ | 35.65(1.67)^A^ | 0.0003 |
| Born in Australia (%)^5^ | 66.95(2.7)^A^ | 69.6(1.86)^A^ | 68.98(1.86)^A^ | 67.31(1.59)^A^ | 0.6835 |
| Inner regional living in Australia (%)^5^ | 16.86(2.02)^A^ | 16.94(1.51)^A^ | 19.09(1.86)^A^ | 18.92(1.43)^A^ | 0.2146 |
| Lowest 10% SEIFA ranking (%)^5^ | 11.99(1.86)^A^ | 7.95(1.26)^AB^ | 7.14(0.95)^B^ | 9.87(1.19)^AB^ | 0.1835 |
| **UPF whole-grain consumers^1^** | | | | | |
| n (unweighted) | 1457 | 1139 | 1223 | 1283 | - |
| Median whole-grain intake (g/10MJ/day)(IQR) | 4.8(0, 38.42) | 11.11(3.49, 38.27) | 32.8(21.9, 56.3) | 71.56(50.74, 96.3) | - |
| Median non UPF whole-grain intake (g/10MJ/day)(IQR) | 4.8(0, 38.42) | 6.76(0, 31.61) | 11.95(0, 34.89) | 7.29(0, 31.91) | - |
| Median UPF whole-grain intake (g/10MJ/day)(IQR) | 0 | 3.35(1.42, 6.72) | 21.02(16.13, 26.45) | 52.04(40.96, 71.05) | - |
| Median refined grain intake (g/10MJ/day)(IQR) | 108.9(71.43-154.6) | 124.5(86.55-163.9) | 105.6(73.28-140.6) | 94.71(63.49-134.0) | - |
| Age^3,4^ | 45.67(0.58)^AB^ | 44.08(0.65)^A^ | 46.89(0.68)^B^ | 49.9(0.75) | <0.0001 |
| Female (%)^5^ | 49.8(1.66)^A^ | 49.26(1.8)^A^ | 48.25(1.66)^A^ | 45.93(1.72)^A^ | 0.4252 |
| Energy intake (kJ/day)^3,8^ | 8147.2(102.7)^A^ | 9092.2(110.6)^B^ | 8844.4(108.0)^B^ | 8290.3(105.4)^A^ | 0.6766 |
| Fibre intake (g/day)^3,9^ | 20.87(0.24) | 21.97(0.26)^A^ | 23.06(0.3)^A^ | 26.99(0.35) | <0.0001 |
| Sodium intake (mg/day)^3,9^ | 2419.5(31.86)^A^ | 2453.3(35.2)^A^ | 2339.0(27.7)^A^ | 2334.12(29.55)^A^ | 0.0281 |
| Saturated fat intake (g/day)^3,9^ | 27.7(0.38)^A^ | 27.73(0.39)^A^ | 27.78(0.28)^A^ | 26.1(0.31) | 0.0008 |
| Polyunsaturated fat intake (g/day)^3,9^ | 11.81(0.22)^A^ | 11.71(0.24)^A^ | 11.19(0.17)^A^ | 11.16(0.23)^A^ | 0.0072 |
| Monounsaturated fat intake (g/day)^3,9^ | 29.3(0.39)^B^ | 29.16(0.43)^B^ | 27.55(0.25)^A^ | 26.41(0.32)^A^ | <0.0001 |
| Trans fat intake (mg/day)^3,9^ | 1394.4(28.52)^A^ | 1337.6(28.38)^A^ | 1372.3(24.5)^A^ | 1308.9(24.13)^A^ | 0.0496 |
| Free sugar intake (% energy)^3,8^ | 10.07(0.32)^A^ | 9.88(0.27)^A^ | 10.43(0.25)^A^ | 8.85(0.21) | 0.0173 |
| Alcohol intake (g/day)^3,9^ | 17.3(1.04)^B^ | 14.21(0.96)^AB^ | 13.28(0.8)^A^ | 10.29(0.99)^A^ | <0.0001 |
| University graduate (%)^5^ | 17.23(1.63)^A^ | 22.78(1.74)^A^ | 20.57(1.45)^A^ | 17.22(1.56)^A^ | 0.2510 |
| Current smoker daily (%)^5^ | 21.36(1.62) | 12.96(1.42)^A^ | 13.67(1.36)^A^ | 9.31(0.83)^A^ | <0.0001 |
| Low physical activity level (%)^5^ | 35.67(1.74)^A^ | 39.25(1.86)^B^ | 32.98(2.06)^AB^ | 34.45(1.93)^A^ | 0.0140 |
| Born in Australia (%)^5^ | 64.9(2.07)^A^ | 68.43(2.32)^A^ | 71.49(1.99)^A^ | 69.51(1.71)^A^ | 0.1040 |
| Inner regional living in Australia (%)^5^ | 17.21(1.57)^A^ | 18.8(1.52)^A^ | 17.72(1.58)^A^ | 18.94(1.53)^A^ | 0.7054 |
| Lowest 10% SEIFA ranking (%)^5^ | 9.97(1.17)^A^ | 8.38(1.65)^A^ | 7.23(0.93)^A^ | 9.48(1.4)^A^ | 0.5253 |
| **Non-UPF whole-grain consumers^1^** | | | | | |
| n (unweighted) | 2305 | 912 | 921 | 964 | - |
| Median whole-grain intake (g/10MJ/day)(IQR) | 7.49(0, 32.41) | 24.84(13.72, 43.01) | 45.71(34.73, 68.12) | 83.43(64.85, 108.7) | - |
| Median non UPF whole-grain intake (g/10MJ/day)(IQR) | 0 | 11.72(7.92, 16.51) | 31.44(26.16, 37.81) | 65.44(53.67, 85.01) | - |
| Median UPF whole-grain intake (g/10MJ/day)(IQR) | 7.49(0, 32.41) | 12.28(0.75, 31.35) | 12.93(0, 35.52) | 6.32(0, 26.47) | - |
| Median refined grain intake (g/10MJ/day)(IQR) | 121.0(81.61-160.7) | 111.1(77.71-154.1) | 99.53(68.0-135.8) | 88.5(56.49-122.8) | - |
| Age^3,4^ | 44.78(0.47)^A^ | 45.37(0.71)^A^ | 47.12(0.8)^A^ | 51.77(0.77) | <0.0001 |
| Female (%)^5^ | 43.5(1.37)^A^ | 55.55(2.68)^B^ | 49.98(2.13)^AB^ | 51.77(2.12)^B^ | 0.0003 |
| Energy intake (kJ/day)^3,8^ | 8285.7(71.8)^A^ | 9215.4(113.6) | 8651.7(132.3)^A^ | 8559.1(101.5)^A^ | 0.5538 |
| Fibre intake (g/day)^3,9^ | 20.32(0.21) | 22.75(0.32) | 25.23(0.36) | 28.26(0.3) | <0.0001 |
| Sodium intake (mg/day)^3,9^ | 2457.7(24.31)^B^ | 2436.3(28.57)^B^ | 2324.8(41.4)^AB^ | 2230.5(37.92)^A^ | <0.0001 |
| Saturated fat intake (g/day)^3,9^ | 28.46(0.28)^C^ | 27.51(0.42)^BC^ | 26.6(0.42)^AB^ | 25.15(0.33)^A^ | <0.0001 |
| Polyunsaturated fat intake (g/day)^3,9^ | 10.73(0.16)^A^ | 11.45(0.27)^AB^ | 12.2(0.22)^BC^ | 12.7(0.26)^C^ | <0.0001 |
| Monounsaturated fat intake (g/day)^3,9^ | 28.15(0.31)^A^ | 28.57(0.37)^A^ | 28.67(0.46)^A^ | 27.25(0.41)^A^ | 0.1521 |
| Trans fat intake (mg/day)^3,9^ | 1429.1(21.93)^B^ | 1385.1(31.29)^B^ | 1272.7(28.34)^A^ | 1225.0(26.18)^A^ | <0.0001 |
| Free sugar intake (% energy)^3,8^ | 10.78(0.17) | 9.7(0.31)^B^ | 9.2(0.33)^AB^ | 8.19(0.26)^A^ | <0.0001 |
| Alcohol intake (g/day)^3,9^ | 15.37(0.75)^B^ | 15.87(1.24)^B^ | 12.23(0.84)^AB^ | 10.19(0.92)^A^ | <0.0001 |
| University graduate (%)^5^ | 17.44(1.21)^A^ | 19.42(1.62)^A^ | 22.17(1.8)^A^ | 21.17(1.85)^A^ | 0.0123 |
| Current smoker daily (%)^5^ | 19.29(1.14) | 10.86(1.29)^A^ | 12.16(1.76)^A^ | 9.5(1.42)^A^ | 0.0001 |
| Low physical activity level (%)^5^ | 38.02(1.36)^A^ | 28.21(1.9)^B^ | 36.41(2.25)^AB^ | 36.14(2.24)^B^ | 0.0001 |
| Born in Australia (%)^5^ | 69.36(1.35)^A^ | 69.71(2.6)^A^ | 66.39(2.43)^A^ | 66.76(2.04)^A^ | 0.5135 |
| Inner regional living in Australia (%)^5^ | 18.43(1.38)^AB^ | 14.6(1.64)^A^ | 21.38(1.98)^B^ | 17.62(2.05)^AB^ | 0.0388 |
| Lowest 10% SEIFA ranking (%)^5^ | 9.9(1.12)^A^ | 5.95(1.18)^A^ | 7.54(1.39)^A^ | 10.3(1.41)^A^ | 0.3036 |

1 N (weighted) is based on participants 19 years and over within the NNPAS 2011-12 that have complete information for all characteristics of interest
2 P-value for linear trend. A significance is determined at *p*<0.05
3 Reported as x̅ (SEM)
4 Linear regression adjusted for sex
5 Reported as percentage (SE)
6 Linear regression adjusted for age and sex
7 Linear regression adjusted for age, sex, and energy intake
8 Linear regression adjusted for age, sex, and non-ultra-processed and ultra-processed whole-grain intake simultaneously
9 Linear regression adjusted for age, sex, energy intake, and non-ultra-processed and ultra-processed whole-grain intake simultaneously

*Non-consumers and tertiles sharing capital letters within rows are not statistically significant from each other. Comparison of means were conducted through pairwise comparison. Comparison of percentages were conducted through individual Pearson’s chi^2^ analysis. All comparisons applied a Bonferroni correction for multiple comparisons such that a significant difference was observed at P<0.008.*

**Supplementary Material 6** Population characteristics of non-consumers and consumers of whole grains by level of processing and in US adults (n 6836).

|  | **Tertiles of whole-grain intake (g/10MJ/d)** | | | | |
| --- | --- | --- | --- | --- | --- |
|  | **Non consumers** | **T1** | **T2** | **T3** | p-value^2^ |
| **Total whole-grain consumers^1^** | | | | | |
| n (unweighted) | 2239 | 1532 | 1533 | 1532 |  |
| Median whole-grain intake (g/10MJ/day)(95% CI) | 0 | 7.66(7.06, 8.27) | 24.9(23.99, 25.81) | 61.44(58.57, 64.32) |  |
| Median non UPF whole-grain intake (g/10MJ/day)(95% CI) | 0 | 0(-3.38, 3.38) | 0(-4.7, 4.7) | 0(-8.56, 8.56) |  |
| Median UPF whole-grain intake (g/10MJ/day)(95% CI) | 0 | 7.1(6.4, 7.79) | 22.74(21.98, 23.49) | 45.15(42.67, 47.63) |  |
| Median refined grain intake (g/day)(95% CI) | 110.9(106.3, 115.5) | 109.3(103.6, 115.0) | 100.4(95.6, 105.2) | 79.97(75.16, 84.79) |  |
| Age^3,4^ | 45.19(0.55) | 48.12(0.67)^A^ | 49.22(0.79)^A^ | 50.5(0.93)^A^ | 0.0001 |
| Female (%)^5^ | 48.23(1.47)^A^ | 49.62(2.09)^AB^ | 54.65(1.95)^AB^ | 54.3(1.67)^B^ | 0.0213 |
| Energy intake (kJ/day)^3,6^ | 8313.9(111.4)^A^ | 9399.5(148.6)^B^ | 9067.1(130.5)^B^ | 8193.0(123.1)^A^ | 0.0010 |
| Fibre intake (g/day)^3,7^ | 13.57(0.28)^A^ | 14.45(0.28)^A^ | 17.61(0.29) | 22.11(0.35) | <0.0001 |
| Sodium intake (mg/day)^3,7^ | 3369.2(28.2)^A^ | 3420.9(50.3)^A^ | 3355.9(35.7)^A^ | 3288.2(31.6)^A^ | 0.0140 |
| Saturated fat intake (g/day)^3,7^ | 27.06(0.29)^A^ | 27.24(0.34)^A^ | 26.94(0.36)^A^ | 25.01(0.38) | <0.0001 |
| Polyunsaturated fat intake (g/day)^3,7^ | 19.02(0.33)^A^ | 19.52(0.25)^A^ | 18.65(0.32)^A^ | 19.04(0.26)^A^ | 0.5505 |
| Monounsaturated fat intake (g/day)^3,7^ | 28.74(0.25)^AB^ | 28.96(0.38)^A^ | 28.05(0.32)^AB^ | 27.56(0.37)^B^ | 0.0017 |
| Added sugar intake (g/day)^3,7^ | 15.79(0.52)^A^ | 15.14(0.55)^AB^ | 14.46(0.5)^AB^ | 13.69(0.43)^B^ | 0.0038 |
| Moderate alcohol consumption (%)^5^ | 63.89(1.68)^A^ | 68.65(1.99)^A^ | 65.54(2.09)^AB^ | 63.05(2.28)^B^ | 0.0010 |
| >12 years education (%)^5^ | 55.95(2.35)^A^ | 61.44(2.71)^A^ | 70.68(1.94)^B^ | 74.72(1.91)^B^ | <0.0001 |
| Current smoker (%)^5^ | 22.88(1.36)^A^ | 19.27(1.67)^A^ | 13.54(1.48)^AC^ | 9.61(1.31)^BC^ | <0.0001 |
| Pack years smoking^3^ | 8.12(0.63)^A^ | 7.67(0.69)^AB^ | 6.68(0.67)^AB^ | 5.66(0.52)^B^ | 0.0009 |
| Physically active (%)^5^ | 64.44(1.79)^A^ | 69.42(1.93)^AB^ | 70.21(1.54)^B^ | 71.41(1.59)^B^ | 0.0102 |
| MET score^3,8^ | 4289.6(239.0)^A^ | 4301.1(277.0)^A^ | 4053.7(254.7)^A^ | 3823.4(253.5)^A^ | 0.1562 |
| Born in America (%)^5^ | 81.21(1.8)^A^ | 86.19(1.45)^B^ | 85.24(1.59)^AB^ | 83.76(1.64)^AB^ | 0.0240 |
| Non-Hispanic White (%)^5^ | 58.64(2.77) | 68.35(2.66)^A^ | 68.3(2.91)^AB^ | 69.54(2.74)^B^ | <0.0001 |
| <1.3 PIR (%)^5^ | 26.36(1.47) | 16.39(1.32)^A^ | 17.09(1.39)^A^ | 16.15(1.78)^A^ | <0.0001 |
| **UPF whole-grain consumers^1^** | | | | | |
| n (unweighted) | 2584 | 1417 | 1418 | 1417 |  |
| Median whole-grain intake (g/10MJ/day)(95% CI) | 0 | 7.19(6.52, 7.85) | 21.73(21.04, 22.41) | 51.51(47.57, 55.45) |  |
| Median non UPF whole-grain intake (g/10MJ/day)(95% CI) | 0 | 0(-5.35, 5.35) | 0(-6.8, 6.8) | 0(-6.17, 6.17) |  |
| Median UPF whole-grain intake (g/10MJ/day)(95% CI) | 0 | 6.19(5.75, 6.64) | 20.08(19.59, 20.57) | 45.35(42.88, 47.81) |  |
| Median refined grain intake (g/day)(95% CI) | 109.3(104.2, 114.4) | 108.7(103.2, 114.2) | 100.3(95.17, 105.5) | 85.69(80.37, 91.01) |  |
| Age^3,4^ | 45.59(0.56) | 47.68(0.68)^A^ | 48.54(0.62)^A^ | 51.64(0.99) | <0.0001 |
| Female (%)^5^ | 49.12(1.34)^A^ | 51.52(1.98)^AB^ | 51.47(1.98)^AB^ | 55.06(1.99)^B^ | 0.1312 |
| Energy intake (kJ/day)^3,9^ | 8434.2(139.5)^A^ | 9483.5(199.1)^B^ | 9149.4(170.9)^B^ | 8353.5(128.6)^A^ | 0.0369 |
| Fibre intake (g/day)^3,10^ | 17.56(0.39)^A^ | 18.04(0.4)^A^ | 20.2(0.37) | 24.66(0.55) | <0.0001 |
| Sodium intake (mg/day)^3,10^ | 3333.3(39.5)^A^ | 3396.0(53.6)^A^ | 3325.3(46.08)^A^ | 3268.9(41.1)^A^ | 0.0770 |
| Saturated fat intake (g/day)^3,10^ | 25.47(0.42)^A^ | 25.69(0.47)^A^ | 25.61(0.51)^AB^ | 24.23(0.51)^B^ | 0.0007 |
| Polyunsaturated fat intake (g/day)^3,10^ | 19.42(0.36)^A^ | 20.01(0.27)^A^ | 19.34(0.49)^A^ | 19.14(0.33)^A^ | 0.2527 |
| Monounsaturated fat intake (g/day)^3,10^ | 28.52(0.32)^A^ | 28.8(0.36)^A^ | 28.21(0.47)^AB^ | 27.32(0.41)^B^ | 0.0017 |
| Added sugar intake (g/day)^3,10^ | 14.5(0.64)^A^ | 14.43(0.53)^A^ | 13.52(0.54)^A^ | 13.43(0.65)^A^ | 0.0658 |
| Moderate alcohol consumption (%)^5^ | 63.82(1.65)^A^ | 67.95(1.72)^A^ | 67.81(2.25)^AB^ | 62.14(2.2)^B^ | 0.0019 |
| >12 years education (%)^5^ | 57.54(2.2) | 63.69(2.77) | 69.67(2.22)^A^ | 72.91(2.08)^A^ | <0.0001 |
| Current smoker (%)^5^ | 22.69(1.48)^A^ | 17.61(1.71)^AB^ | 14.33(1.41)^BC^ | 9.51(1.16)^C^ | <0.0001 |
| Pack years smoking^3^ | 7.86(0.58)^A^ | 7.43(0.73)^AB^ | 7.26(0.64)^AB^ | 5.52(0.51)^B^ | 0.0023 |
| Physically active (%)^5^ | 64.79(1.68)^A^ | 69.32(1.89)^AB^ | 71.14(1.5)^B^ | 71.02(2.05)^AB^ | 0.0150 |
| MET score^3,8^ | 4329.3(196.8)^A^ | 4094.7(268.2)^A^ | 4190.3(265.5)^A^ | 3744.5(237.1)^A^ | 0.1297 |
| Born in America (%)^5^ | 80.06(1.78)^A^ | 84.54(1.75)^AB^ | 87.07(1.3)^B^ | 86.11(1.56)^B^ | <0.0001 |
| Non-Hispanic White (%)^5^ | 57.51(2.91) | 66.02(2.8) | 69.79(2.52)^A^ | 73.88(2.7)^A^ | <0.0001 |
| <1.3 PIR (%)^5^ | 25.15(1.36) | 16.25(1.37)^A^ | 17.62(1.21)^A^ | 15.74(1.47)^A^ | <0.0001 |
| **Non-UPF whole-grain consumers^1^** | | | | | |
| n (unweighted) | 5694 | 380 | 381 | 381 |  |
| Median whole-grain intake (g/10MJ/day)(95% CI) | 7.99(6.03, 9.95) | 23.57(18.04, 29.1) | 49.18(44.72, 53.63) | 83.41(76.46, 90.36) |  |
| Median non UPF whole-grain intake (g/10MJ/day)(95% CI) | 0 | 12.19(10.28, 14.09) | 29.75(28.25, 31.25) | 63.07(58.48, 67.66) |  |
| Median UPF whole-grain intake (g/10MJ/day)(95% CI) | 7.99(6.03, 9.95) | 11.79(6.15, 17.43) | 17.27(13.01, 21.53) | 11.19(6.34, 16.04) |  |
| Median refined grain intake (g/day)(95% CI) | 104.5(101.6, 107.4) | 95.72(86.81, 104.6) | 85.58(76.67, 94.49) | 67.72(57.29, 78.14) |  |
| Age^3,4^ | 47.9(0.51)^A^ | 49.36(1.5)^A^ | 49.29(1.07)^A^ | 46.84(1.67)^A^ | 0.9518 |
| Female (%)^5^ | 51.18(0.98)^A^ | 48.69(3.95)^AB^ | 51.48(3.12)^AB^ | 60.31(2.4)^B^ | 0.0874 |
| Energy intake (kJ/day)^3,9^ | 8745.5(69.1)^A^ | 9634.7(279.1)^C^ | 9019.1(233.0)^AC^ | 8021.3(258.9)^B^ | 0.1728 |
| Fibre intake (g/day)^3,10^ | 16.12(0.18) | 19.54(0.55)^A^ | 21.0(0.69)^AB^ | 23.81(0.95)^B^ | <0.0001 |
| Sodium intake (mg/day)^3,10^ | 3367.7(23.2)^A^ | 3359.1(54.1)^A^ | 3348.0(77.2)^A^ | 3248.7(45.6)^A^ | 0.0231 |
| Saturated fat intake (g/day)^3,10^ | 26.95(0.21)^A^ | 24.56(0.67)^B^ | 25.99(0.87)^AB^ | 23.52(0.67)^B^ | <0.0001 |
| Polyunsaturated fat intake (g/day)^3,10^ | 18.94(0.16)^A^ | 20.93(0.72)^A^ | 19.3(0.49)^A^ | 18.74(0.49)^A^ | 0.5900 |
| Monounsaturated fat intake (g/day)^3,10^ | 28.35(0.19)^A^ | 28.34(0.64)^A^ | 29.1(0.83)^A^ | 27.06(0.55)^A^ | 0.1791 |
| Added sugar intake (g/day)^3,10^ | 15.01(0.7)^A^ | 14.41(1.28)^A^ | 12.76(1.15)^A^ | 13.69(1.14)^A^ | 0.0319 |
| Moderate alcohol consumption (%)^5^ | 65.11(1.26)^AB^ | 62.52(4.27)^B^ | 73.75(3.92)^A^ | 60.81(4.09)^AB^ | 0.1971 |
| >12 years education (%)^5^ | 62.73(1.5)^A^ | 74.47(4.8)^AB^ | 79.58(2.78)^B^ | 75.95(4.31)^B^ | <0.0001 |
| Current smoker (%)^5^ | 17.71(0.95)^A^ | 14.5(4.23)^AB^ | 8.94(2.16)^BC^ | 13.22(2.76)^AC^ | 0.0290 |
| Pack years smoking^3^ | 7.4(0.37)^A^ | 5.22(0.94)^A^ | 5.68(0.83)^A^ | 6.23(1.22)^A^ | 0.0657 |
| Physically active (%)^5^ | 68.01(1.01)^A^ | 72.43(3.52)^A^ | 71.6(2.84)^A^ | 69.73(3.01)^A^ | 0.3641 |
| MET score^3,8^ | 4211.6(148.4)^A^ | 3758.7(493.6)^A^ | 3772.4(448.2)^A^ | 3896.4(443.7)^A^ | 0.2285 |
| Born in America (%)^5^ | 84.71(1.16)^A^ | 85.62(2.12)^A^ | 80.37(2.85)^AB^ | 71.19(3.79)^B^ | <0.0001 |
| Non-Hispanic White (%)^5^ | 66.49(0.7)^A^ | 68.48(3.83)^AB^ | 59.72(4.75)^BC^ | 53.8(4.77)^C^ | <0.0001 |
| <1.3 PIR (%)^5^ | 20.38(1.07)^A^ | 10.41(1.88)^B^ | 12.46(1.99)^BC^ | 24.33(5.0)^AC^ | 0.0012 |

1 N (weighted) is based on participants 20 years and over within the NHANES 2015-18 that have complete information for all characteristics of interest
2 P-value for linear trend. A significance is determined at *p*<0.05
3 Reported as x̅ (SEM)
4 Linear regression adjusted for sex
5 Reported as percentage (SE)
6 Linear regression adjusted for age and sex
7 Linear regression adjusted for age, sex, and energy intake
8 Linear regression adjusted for age, sex, and ethnicity
9 Linear regression adjusted for age, sex, and non-ultra-processed and ultra-processed whole-grain intake simultaneously
10 Linear regression adjusted for age, sex, energy intake, and non-ultra-processed and ultra-processed whole-grain intake simultaneously

*Non-consumers and tertiles sharing capital letters within rows are not statistically significant from each other. Comparison of means were conducted through pairwise comparison. Comparison of percentages were conducted through individual Pearson’s chi^2^ analysis. All comparisons applied a Bonferroni correction for multiple comparisons such that a significant difference was observed at P<0.008.*
